# HopPER: an adaptive model for probability estimation of influenza reassortment through host prediction

**DOI:** 10.1186/s12920-019-0656-7

**Published:** 2020-01-23

**Authors:** Rui Yin, Xinrui Zhou, Shamima Rashid, Chee Keong Kwoh

**Affiliations:** School of Computer Science and Engineering, Nanyang Technological University, 50 Nanyang Avenue, Singapore, 639798 Singapore

**Keywords:** Influenza, Reassortment estimation, Host tropism, Random forest

## Abstract

**Background:**

Influenza reassortment, a mechanism where influenza viruses exchange their RNA segments by co-infecting a single cell, has been implicated in several major pandemics since 19th century. Owing to the significant impact on public health and social stability, great attention has been received on the identification of influenza reassortment.

**Methods:**

We proposed a novel computational method named HopPER (Host-prediction-based Probability Estimation of Reassortment), that sturdily estimates reassortment probabilities through host tropism prediction using 147 new features generated from seven physicochemical properties of amino acids. We conducted the experiments on a range of real and synthetic datasets and compared HopPER with several state-of-the-art methods.

**Results:**

It is shown that 280 out of 318 candidate reassortants have been successfully identified. Additionally, not only can HopPER be applied to complete genomes but its effectiveness on incomplete genomes is also demonstrated. The analysis of evolutionary success of avian, human and swine viruses generated through reassortment across different years using HopPER further revealed the reassortment history of the influenza viruses.

**Conclusions:**

Our study presents a novel method for the prediction of influenza reassortment. We hope this method could facilitate rapid reassortment detection and provide novel insights into the evolutionary patterns of influenza viruses.

## Background

Influenza A viruses, as highly infectious respiratory pathogens, are able to evade host immune responses and transmit across host species. A complete influenza genome consists of eight independent gene segments, where the subtype of influenza is characterized by the surface glycoproteins hemagglutinin (HA) and neuraminidase (NA) [1]. Transcription and replication take place by the viral RNA-dependent polymerase complex polymerase acidic protein (PA), polymerase basic protein 1 (PB1) and polymerase basic protein 2 (PB2) [2]. The rest of the segments encode the nucleoprotein (NP), matrix protein (M1), ion channel protein (M2) and two non-structural proteins (NS1 and NS2). This structure of the virus allows for the exchange of eight RNA segments between influenza viruses coinfecting a cell [3]. The process of genetic recombination, named reassortment, may lead to the emergence of novel progeny viruses [4].

It has been well recognized that reassortment is an evolutionary mechanism of segmented viruses that play an important role in the interspecies transmission and generating novel strains of influenza. The reassortment could accelerate the rate of acquiring new genetic markers that would faster overcome host barriers than the slow process of incremental accumulation of mutations [5]. Three of the four major influenza pandemics occurred since the 19th century were due to the reassortment that produced new strains infecting humans [6]. The evidence indicated that the HA and NA segments of the 1957 Asian pandemic were replaced by gene segments related to avian strains. The reassortment of human and avian strains with an H3 HA gene derived from avian-origin viruses led to the 1968 H3N2 pandemic [7]. In addition, reassortment between two different swine influenza viruses, which themselves contained genes from previous human, swine and avian influenza strains, caused another pandemic in 2009 [8]. These pandemics have not only killed numerous people but also led to enormous economic losses. Therefore, early identification of influenza reassortment and potential reassortant strains are crucial for the surveillance and prevention of pandemics in the future.

With the rapid growth of flu data in recent years, increasing complete influenza genomes are publicly available [9]. There is little concern about the acquisition and interpretation of the data. Many efforts have been made to detect influenza reassortment events using the influenza genomic data. The common approach of identifying influenza reassortment is to construct fixed phylogenetic trees relating each segment of the strains [10–12]. Two methods were proposed for identifying reassortment events based on the difference between phylogenetic trees or tree subsets [13]. These trees are compared to detect disagreements of different strains, but it is a laborious and time-consuming process. Moreover, it provides no guarantee that all reassortments have been found. To account for the uncertainty in the inferred phylogenies, a novel computational method named GiRaF was developed to identify reassortment [14]. In GiRaF, large collections of Markov chain Monte Carlo sampled trees were searched for groups of incompatible splits by a fast biclique enumeration algorithm. This successfully detected some known reassortments in avian, human and swine influenza strains. Yurovsky and Moret presented a fully automated flu reassortment finder called FluRF that employed a bottom-up search on the reconstructed phylogenetic trees of full and segment-based genomes [15]. However, the computational cost of phylogeny laid a formidable barrier for reassortment detection using phylogenetic analysis with a large scale of the dataset. Silva et al. aimed to solve this problem by formulating a phylogeny independent method that only utilized nucleotide distance matrices as input for reassortment detection [16]. Furthermore, Rabadan et al. provided a quantitative method to measure the genetic shift from nucleotide sequence data that did not rely on phylogenetic analysis for reassortment detection [17]. Villa and Lässig determined rate and average selective effect of reassortment process in human influenza H3N2 using a new method to map reassortment events from joint genealogies of multiple genome segments [18]. Eng et al. developed an influenza reassortment simulation tool through host tropism protein signatures [19]. This program computationally simulates reassortment between the eight viral segments and then generates a list of all possible reassortant progeny based on the signatures.

Despite the growing data of genomic sequences and powerful computational capability for constructing various phylogenies to detect reassortment events, these approaches are generally applicable in a small scale of the dataset with well-defined phylogenetic trees. In particular, none of the existing approaches scale well to large datasets in detecting all reassortants. In this paper, we develop a novel approach named HopPER (Host-prediction-based Probability Estimation of Reassortment) that employs machine learning techniques to calculate the reassortment probability by predicting the host tropism in a given collection of genomic sequences. HopPER first generates the feature vectors by seven physicochemical properties of amino acids from influenza sequences of three major hosts (avian, human, swine) with global descriptors CTD (Composition, Transition and Distribution). It then applies a kernel perspective on host probability estimation by the random forest [20] for a single sequence and then combines all segments of the genome to produce an overall estimation of reassortment probability. We tested HopPER on both real datasets and synthetic datasets to evaluate the capacity of estimating the reassortment possibility of genomes. HopPER is compared with some state-of-the-art methods. The results show that HopPER has successfully identified reassortments with high precision.Furthermore, HopPER is efficient in detecting reassortment for even incomplete genomes (with at least two available genomic segments) and in analyzing large datasets. We hope HopPER can assist flu surveillance and prevent future pandemics.

## Methods

### Problem formulation

**Fig. 1:**
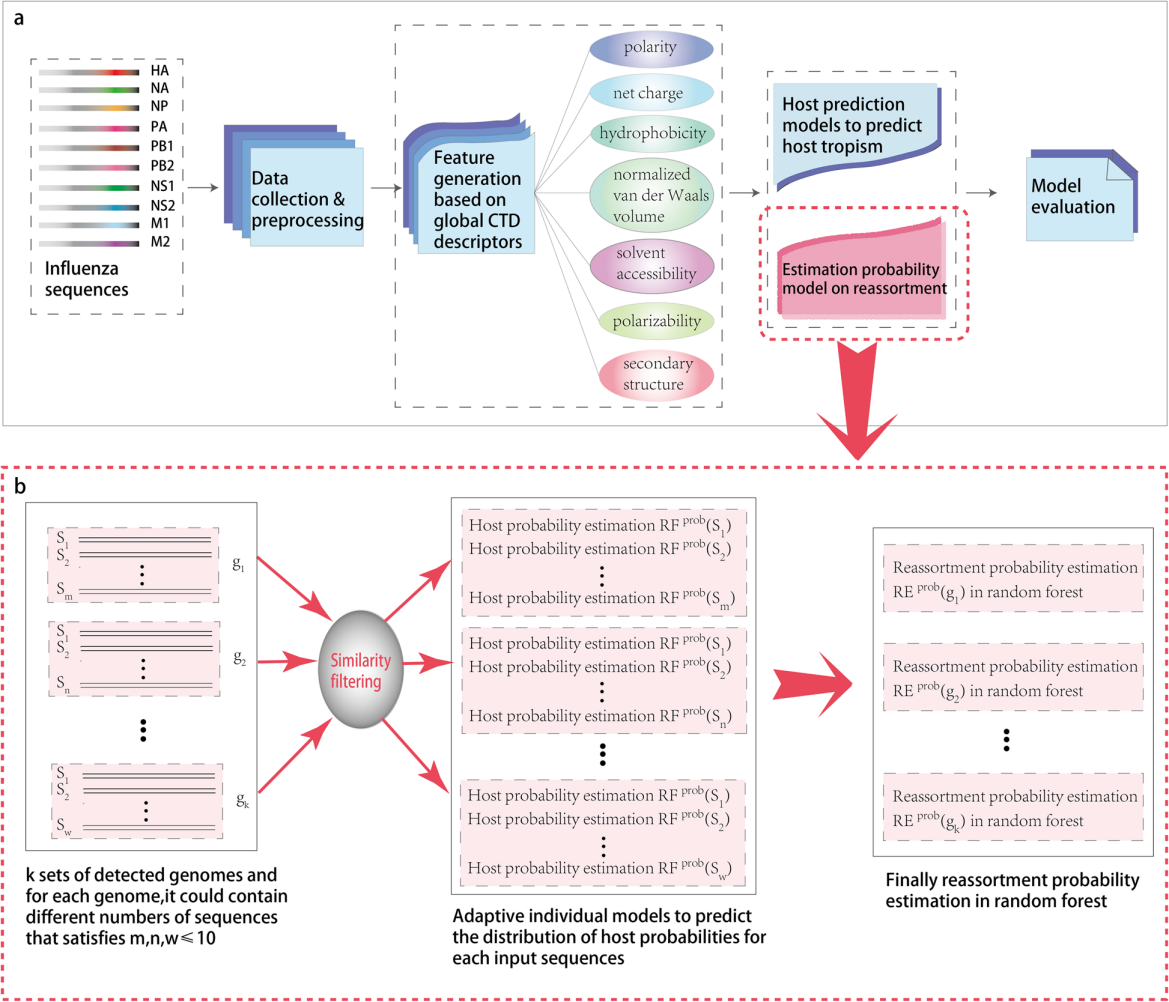
Schematic overview of analysis workflow in HopPER. **a** The general diagram of the host prediction model based on seven physicochemical properties and reassortment probability estimation in the random forest. **b** Specific algorithmic steps for estimation probability model on influenza genome reassortment detection

The concepts of reassortment are broadly applicable to other multipartite genomes, most of which have been studied. Here, our interest is only influenza reassortment. As far as we know, the reassortant strains are responsible for the majority of flu pandemics in history and will continuously threaten public health. While any exchange of genetic material between different influenza viral RNA segments can be considered as reassortment. In this paper, we mainly focus on identifying interspecies reassortments that have occurred across hosts. It is similar to definitions of host tropism predictors in the literature, except that here the problem is formulated probabilistically to enable a quantified estimate of host origin. Hence, host tropism is modelled by quantifying the reassortant probabilities. The model can also detect intra-host reassortments, for instance between different viral strains that have originate from one single host category such as avian. In the model, the actual host in which the mixing occurred is disregarded and the focus is mainly on detecting past reassortants and the potential evolutionary relationships that may be inferred. For all practical purposes, we only use avian, human and swine strains that account for the overwhelming majority of the existing sequence data. The following subsections respectively elaborate the dataset and the structure of the model. Figure 1 presents the flowchart of HopPER.

### Data collection and preprocessing

The amino acid sequences of all segments with avian, human and swine hosts are downloaded from NCBI Influenza Virus Resource [21] on 31 Dec 2017. Only full-length sequences are acquired and duplicate strains are removed from the collection. The results are presented in Table 1. We exclude PB1-F2 and PA-X proteins as they are completely contained in PB1 and PA respectively. It would be impossible for PB1-F2 and PA-X to have different host designation to PB1 and PA. Similarly, segment M consists of M1 and M2 proteins and segment NS comprises NS1 and NS2 proteins. We only select NS1 and M2 proteins as representatives for host tropism prediction. This is because we could collect many more samples on NS1 and M2 to construct the model. Finally, the data of eight different proteins is obtained and we label avian sequences as ’0’, human sequences as ’1’ and swine sequences as ’2’ in the process of host prediction.

**Table 1:** The number of influenza sequences for selected segments on avian, human, swine hosts and combined dataset

Protein	Host type			
	Avian	Human	Swine	Combined
HA	12248	13607	6257	32112
NA	9452	10107	5734	25293
NP	4841	2659	2292	9792
PA	8428	5498	3059	16985
PB1	7699	4869	2892	15460
PB2	8106	5490	2901	16497
NS1	6115	4133	2662	12910
M2	2237	1404	1534	5175

Besides, whole-genome datasets are also collected from NCBI on the same date and settings. To analyze the global patterns of reassortment events from the year 1918 to 2017, we end up with 13598, 20614 and 4380 complete and incomplete genomes of avian, human and swine hosts respectively after data preprocessing. Further analysis is performed to illustrate the potential reassortants using genomic sequences. Also, synthetic genomes are collected from Global Initiative on Sharing All Influenza Data (GISAID) [22]. These strains are synthesized from laboratory and labeled as true reassortants that contains 87 complete genomes and 25 incomplete genomes to validate the performance of our model. The incomplete genomes have at least two different segments so that we could calculate the probability of host tropism for each segment and exert statistical probability estimation to identify the reassortment. Apart from synthetic genomes, we also validate HopPER through real samples studied that have been tested by some state-of-the-art methods. The annotation of real and synthetic genomic samples could be found in Additional file 1: Table S2 and S4.

### Feature transformation

The feature transformation of protein sequences is conducted based on AAindex, a database of amino acid physicochemical properties, substitution matrices and statistical protein contact potentials [23]. We perform the method developed by Dubchak to transform protein sequences into feature vectors [24]. The transformation is implemented by using three global descriptors: composition (C), transition (T) and distribution (D) to calculate the numerical values for each amino acid properties. The amino acid physicochemical properties contain polarity, net charge, hydrophobicity, normalized van der Waals volume, solvent accessibility, polarizability and secondary structure [25]. These amino acids are divided into three different groups based on the physicochemical properties of amino acid indices [26] (Additional file 1: Table S1). The equations for three global descriptors are formulated as follows:

1 $$ Composition = \left(\frac{C_{G1}}{N}, \frac{C_{G2}}{N}, \frac{C_{G3}}{N}\right)  $$

2 $$ Transition = \left(\frac{T_{G1G2}}{N-1}, \frac{T_{G1G3}}{N-1}, \frac{T_{G2G3}}{N-1}\right)  $$

3 $$ Distribution = \left(\frac{D_{i0}}{N}, \frac{D_{i25}}{N}, \frac{D_{i50}}{N}, \frac{D_{i75}}{N}, \frac{D_{i100}}{N}\right)  $$

Composition describes the percentage frequency of each amino acid property groups across the entire protein sequence. *N* is the number of amino acids and *C*_*Gi*_ is the frequency of amino acid property of group *i* in the sequence. Transition characterizes the percentage frequency with which amino acids of a group is followed by another group denoted as *T*_*GiGj*_. It means the property in group *i* is followed by group *j* or the other way around such that *i, j* = 1,2,3 and *G*_*i*_≠*G*_*j*_. The third descriptor illustrates the distribution of each attribute in the sequence and *D*_*i*_ represents the percentage in these positions of the amino acid properties in group *i*. The distribution is based on the first, 25%, 50%, 75% and 100% of the amino acids for each attribute [24]. Therefore, 21, 21 and 105 new features are generated based on seven amino acid physicochemical and structural properties for global CTD descriptor respectively. In total, 147 amino acid feature vectors have been used to build the model for host tropism prediction.

### Host tropism prediction

We first carry out the experiments on the host tropism prediction for selected proteins. The effectiveness of host tropism prediction on influenza HA proteins and zoonotic strains prediction has been demonstrated by Eng et al. [19, 27]. Our previous work supplemented this work on the host prediction of human-adapted subtypes using random forest that achieved better results over other classifiers [28]. By constructing a multitude of decision trees, it applies the general technique of bootstrap aggregating to tree learners and then splits leaf nodes in the trees by random subset of feature space [20]. This comes at the expense of a small increase in the bias and some loss of interpretability, but generally greatly boosts the performance in the final model [29]. To ensure the robustness of our models, all the datasets are split into independent training dataset and testing dataset with a ratio of 0.8:0.2. We first apply ten-fold cross validation technique to develop our models and evaluate the training process with random forest, and then the independent testing dataset is used to assess the ability of our model in predicting the host tropism of new data. The metrics to evaluate the performance include accuracy, precision, recall, G-means [30] and Matthew’s correlation coefficient (MCC) [31].

### Construction of training data

In Fig. 1b, for the given input genomes for reassortment detection, we split the genome into segments. The host tropism prediction for each segment is performed by individual independent models with random forest. To reduce the overfitting of our model for host prediction, we introduce an algorithm named Ratcliff-Obershelp [32] and this method measures the similarity between input sequences and training sequences using gestalt pattern matching. It supports a heuristic that automatically treats certain sequence items as junk and counts how many times each individual item appears in the sequence. The similarity between a pair of sequences ranges from 0 to 1. We set the threshold of 0.99 to filter the sequences from the training data that are similar to input sequences. The remaining sequences are used to train the host prediction model and construct HopPER. Removal of similar sequences establishes independence of train and test datasets. It ensures the cross-validated results are a “true reflection” of model performance. and make our model adaptive to the distinct input genomes for reassortment detection.

### Reassortment probability estimation

In the reassortment probability estimation, we set *x*_*ia*_ as influenza sequence and *y*_*j*_ is the possible host. The variable *x*_*ia*_ represents the influenza protein type *a* in genome *i*. Here, *a* belongs to one of the selected proteins while the ordered elements in set j = 0,1,2 correspond to avian, human and swine hosts, respectively. To better calculate the reassortment probability and make the problem more statistically tractable, it is assumed that the distribution of pairs of influenza sequences and its host labels are independent and identical, that is *x*_*ia*_ and *y*_*j*_ are related according to an unknown conditional class probability function $\mathbb {P}$(*y*_*j*_|*x*_*ia*_). Typical classification is to discriminate whether $\mathbb {P}$(*y*_*j*_|*x*_*ia*_) ≥ 0.5 to predict the class of a new input sequence as described in the “Host tropism prediction” section above. However, our goal is to directly estimate the probability of host tropism for each protein in a genome.

As far as we know, There is no literature regarding reassortment probability estimation in random forest models. This is probably that virologists would usually check for reassortment by a homology search or by phylogenetic analysis of influenza segments. Meanwhile, a previous study has indicated that random forests are difficult to calibrate by standard calibration methods [33]. However, random forest achieves the best performance of estimation among machine learning classifiers after calibration [34]. Some other researchers have investigated the effect of utilizing corrected probability estimates in random forests by Laplace and m-estimates at the nodes have demonstrated its usefulness [35]. Though there still exists limited empirical evidence for the effect of random forest probabilities estimation [36], the framework of kernel regression in the random forest probability estimation produces better results [37].

Consisting of a collection of *T* un-pruned decision trees, where one tree is built from each bootstrap sample, random forests allow consistent estimation of individual probabilities [38]. A tree is constructed by introducing recursive binary splits to the data based on the covariates and only a subset of covariates of predefined size *mtry* is randomly selected at each node. The randomness in each tree is represented by a random variable *θ*∈***Θ***, which is an indicator to index the trees in the forest. The class probability estimates for a terminal node are obtained by the relative frequency of the class in that terminal node. For example, the probability estimate of the tree for a new item is the class probability of the corresponding terminal node. The decision tree will partition the input space by the terminal nodes that would be denoted in the tree generated through *θ*∈***Θ***, where a point *x*_0_ belongs to *R*_*θ*_(*x*_0_). And the number of the samples in this node will be represented by *N*_*θ*_(*x*_0_). Under these assumptions, the probability estimation for a single tree at a point *x*_0_ could be defined as function *f*(*θ*,*x*_0_) formulated below.

4 $$ f(\theta, x_{0}) = \sum_{i=1}^{n} \frac{\prod (x_{i} \in R_{\theta}(x_{0}))y_{j}}{N_{\theta}(x_{0})}  $$

**Fig. 2:**
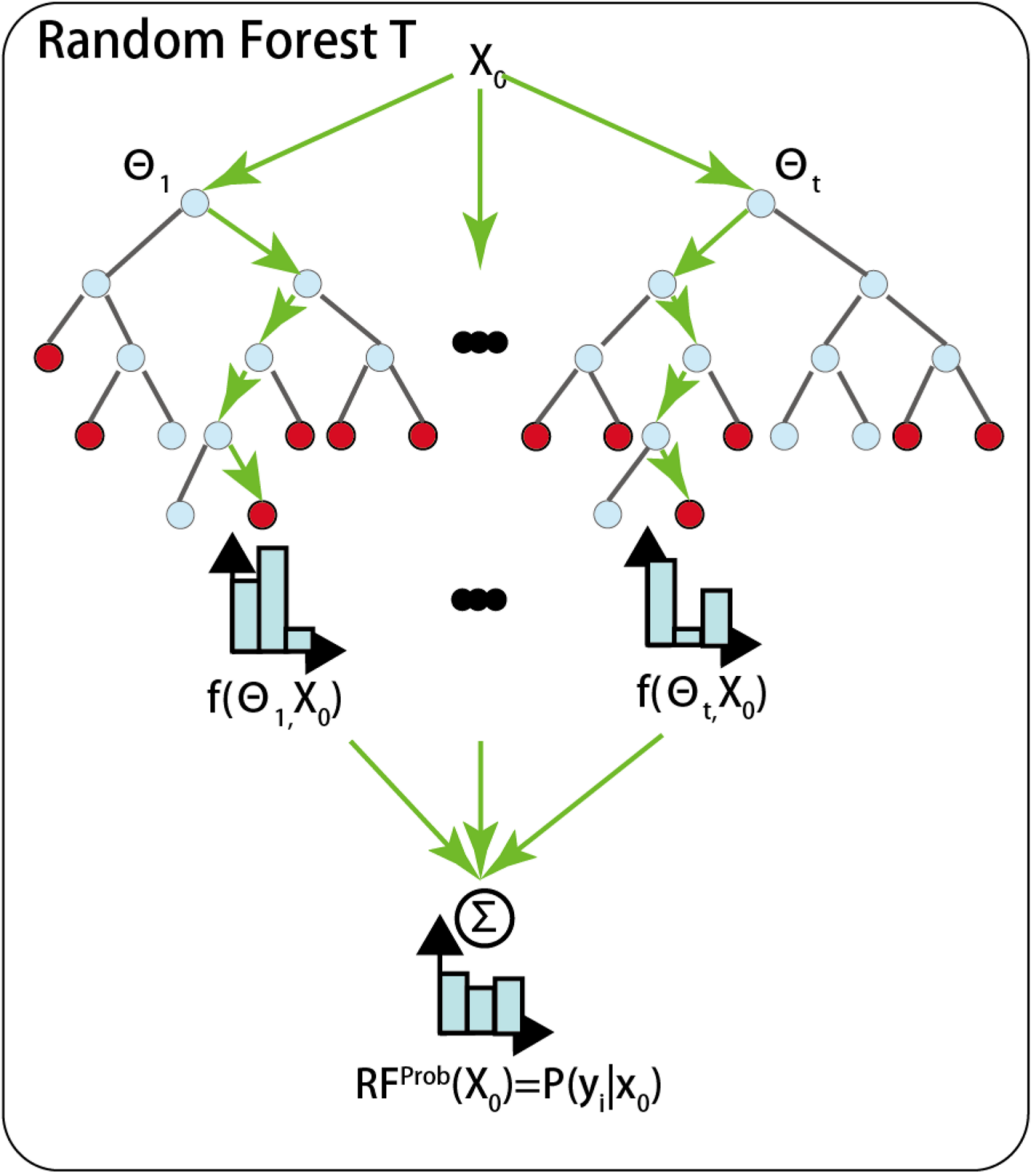
The structure of random forest T for probability estimation. *θ*_*t*_ is an independent random draw and *f*(*θ*_*t*_,*x*_0_) stands for the probability estimate by associated tree *t* at point *x*_0_. $\mathbb {P}$(*y*_*j*_|*x*_0_) characterizes the aggregation of conditional probability of all trees for label *y*_*i*_


A random forest is composed of a set of independent random draws *θ*_1_,...,*θ*_*t*_, and the associated trees *f*(*θ*_1_,·),...,*f*(*θ*_*t*_,·). In the case of host tropism prediction of influenza sequences, we estimate probabilities by making the host label for each tree round(*f*(*θ*_*t*_,*x*_0_)) and counting the fraction of trees that vote for its class. The results are aggregated by averaging the probability estimates denoted by *RF*^*prob*^(·) for the new input data over all trees (Fig. 2). Here we define the function that approximates the conditional class probability $\mathbb {P}$(*y*_*j*_|*x*_0_), calculating the probability of each possible binding host for input sequence *x*_0_, as *RF*^*prob*^(*x*_0_).

5 $$ RF^{prob}(x_{0}) = \frac{1}{T}\sum_{t=1}^{T} round(f(\theta_{t}, x_{0}))  $$

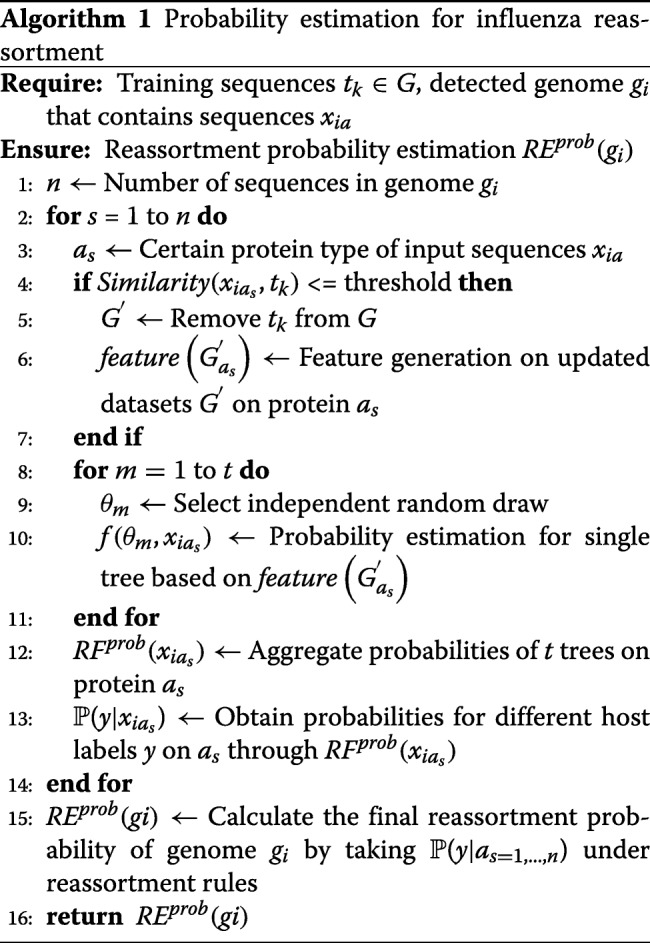


The random forest sustains significant basis for host tropism prediction of influenza sequences but cannot directly identify reassortment or reassortant strains. To perform reassortment probability estimation, we need to know the original host types for all influenza sequences of the genome. In practice, we a set sequence in genome *i* as *x*_*ia*_ where *a*∈*S*{HA, NA, NP, PA, PB1, PB2, NS1, M2 }. The probability estimation for certain host of protein is represented as $x_{ia_{s}}$ and could be calculated by $RF^{prob}(x_{ia_{s}})\phantom {\dot {i}\!}$, that is, $\mathbb {P}$($\phantom {\dot {i}\!}y_{j}|x_{ia_{s}}$), where *y*_*i*_ indicates different host labels and *a*_*s*_ is certain protein. For a candidate genome *g*_*i*_ containing *n* different proteins, we use *a*_*n*_ to denote the possible segments in *g*_*i*_, where *a*_*s*_⊆*a*_*n*_⊆*S* and $x_{ia_{s}} \in g_{i}$. The probability estimation of *g*_*i*_ being non-reassorted could be represented as *NonRE*^*prob*^(*g*_*i*_) and is formulated below.

6 $$ \begin{aligned} NonRE^{prob}(g_{i}) &= P(y_{j}|g_{i}) \\ &=\prod_{}^{a_{n}} P(y_{j}|x_{ia_{s}}) \end{aligned}  $$

Taking all the available sequences into the calculation, the estimate of influenza reassortment probability is given as *RE*^*prob*^(*g*_*i*_) shown in Eq. 7. Algorithm 1 clarifies the detailed steps of estimating reassortment probability. It not only allows the estimation of reassortment probabilities in complete genomes but also displays effectiveness in incomplete genomes. Random forest probability estimation provides a principled way to view the reassortments in terms of conditional probability functions. Hence, such a problem formulation motivates the discussion of estimation function as a fundamental and quantitative way to predict influenza reassortment. For instance, a prediction that an avian host origin is more likely than human or swine host can narrow the sequence or homology search space for a virologist, given a sequence of interest.

7 $$ RE^{prob}(g_{i}) = 1 - \sum_{j=0}^{2} NonRE^{prob}(g_{i})  $$

A genome is regarded as a reassortant strain if the estimated probability is greater than 0.5 by our model, otherwise, it is a non-reassortant strain. We set the true positive value (TPV) in equation (8) to measure the ability of HopPER in reassortant detection. Apart from the detection of reassortment, HopPER can also predict the non-reassortant strains with the same principle. However, as far as we know, the study of non-reassortant strains attract less attention and it is usually difficult to confirm or deny a strain without reassortment. Direct validation of true negative samples by HopPER poses great challenges. As an alternative, we intend to sketch the contours of the distribution of reassortant strains across different years and analyze the rate variation of evolutionary success of viruses generated through reassortment by HopPER. We define the reassortant strain rate (RSR) as the ratio of reassortments that have occurred and the strains reproduced from the past reassortants to total genomic strains, which is a measurement of the subsequent evolutionary success of viruses generated through reassortment. It could be calculated by identifying the reassortant and non-reassortant strains by HopPER. As a result, we could draw the outline of RSR variation by year and analyze the potential evolutionary patterns of the avian, human and swine strains.

8 $$ TPV = \frac{number\,of\,correct\,predictions} {number\,of\,genomes}  $$

## Results and Discussion

### Performance of individual protein on host tropism prediction

After data preprocessing and feature generation for all available sequences from NCBI, prediction models for individual influenza proteins were built by random forest. Table 2 presents the performance of predictive models for individual proteins on independent training and testing data. It is shown that our constructed models achieved outstanding performance in both 10-fold cross validation training data and independent test data. In more details, the HA model obtained the highest accuracy of 0.966 (G-means = 0.953, MCC = 0.943), whereas the lowest was M2 model with 0.876 accuracy (G-means = 0.854, MCC = 0.805) in the training set. Regarding independent test results, our models showed comparative performance with accuracy ranging from 0.865 to 0.965 for different proteins, which further demonstrated the robustness of our proposed models on host tropism prediction. Furthermore, we also reported the predictive performance based on each class of avian, swine and human to help increase the confidence of our models (Additional file 1: Table S3).

**Table 2:** Performance of host tropism predictive models for individual proteins on independent training and testing data

Model	Training data					Testing data				
	Accuracy	Precision	Recall	G-means	MCC	Accuracy	Precision	Recall	G-means	MCC
HA	0.966	0.967	0.956	0.953	0.943	0.965	0.969	0.956	0.955	0.947
NA	0.961	0.962	0.953	0.953	0.939	0.957	0.958	0.95	0.949	0.933
NP	0.947	0.944	0.933	0.931	0.912	0.954	0.951	0.944	0.943	0.927
PA	0.929	0.916	0.893	0.89	0.881	0.922	0.906	0.892	0.888	0.875
PB1	0.931	0.927	0.907	0.902	0.887	0.937	0.933	0.914	0.912	0.898
PB2	0.943	0.937	0.912	0.913	0.906	0.945	0.938	0.923	0.921	0.911
NS1	0.934	0.928	0.917	0.916	0.896	0.931	0.93	0.919	0.917	0.896
M2	0.876	0.866	0.856	0.854	0.805	0.865	0.86	0.853	0.848	0.795

All the prediction models have demonstrated high predictive performance, capable of distinguishing avian, human and swine strains. In the evolutionary history of influenza, the viruses transmit between different host species, which allows for the mixture of gene segments and produces reassortant strains. This might enhance the pathogenicity of the virus, assisting reassortant strains to adapt to new host species [39]. However, it is still a challenge to directly predict the interspecies transmission of influenza viruses and identify the capability of an avian strain to cross the species barrier and infect humans. But the results have proved the effectiveness of all models in predicting host tropism, which paves the way for further reassortment probability estimation through host prediction.

### Evaluation on real datasets

To measure the effectiveness of HopPER, we have applied our model to several independent influenza datasets detected by alternative methods. Genomic sequences for 18 typical reassorted H1N1, H1N2 and H3N2 genomes isolated from pigs in North America were studied in Karasin et al. [40–42]. The datasets of 16 resembled novel 2009 swine-origin isolates, 6 triple reassortant H3N2 strains throughout Canada and 39 reassortment events in swine influenza strains were constructed from a large-scale whole-genome sequences [43–45]. More comprehensively, 36 well supported candidate reassortants [16], 93 single-taxa and multi-taxa reassortment candidates [14, 46] were also selected for validation. A brief description of these genome datasets follows below.

- Karasin et al. investigated the genetic characterization of H1N1, H1N2 and H3N2 viruses that circulated in North America from 1997 to 2005. Due to the occurrence of influenza pandemics in 1957 and 1968, the reassortant human/avian viruses have circulated in the world and been collected from pigs in Europe [47]. After that, the wholly avian H1N1 viruses adapted to the swine population and spread to North America with classical swine influenza virus [48–50]. The results obtained by genetic and phylogenetic trees indicated the evidence for wholly human and reassortant virus genotypes.
- Kingsford et al. dataset contained 16 genome sequences that were similar to swine-origin influenza viruses (S-OIV) that appeared in Thailand. These swine-origin isolates only caused one cause of human infection A/Thailand/271/2005 (H1N1). The comparison between these earlier resembled S-OIV reassortant strains and the real S-OIV strains could facilitate identification of reassortment patterns and may shed light on the cause of S-OIV.
- Olsen et al. dataset contained 6 triple reassortant H3N2 viruses isolated from pigs and turkeys throughout Canada in 2005. These were human classical swine/avian reassortants similar to the viruses in the United States in 1998 except a distinct human-lineage NA segment, which suggested a fast and complicated interspecies transmission of reassortants.
- Khiabanian et al. dataset was used to explore the process and patterns of viral reassortment with 39 complete and incomplete genome sets. The analysis of reassortment phenomena in swine influenza viruses was performed by several statistical techniques. The finding indicated that not only the surface glycoprotein coding proteins (HA and NA) but also the PB1 segment reassorted more frequently compared with other segments in swine viruses.
- de Silva et al. dataset presented 36 well supported candidate reassortants with strong confidence. The results indicated that the introduction of novel HA and/or NA genes into a previous circulating virus led to the reassortment events. Reassortment patterns of the identified strains offered new insight that drove us to draw a more well-rounded picture of the origin of some previously reported reassorted strains.
- Nagarajan et al. presented a more comprehensive reassortment study including human, avian and S-OIV influenza populations. A novel computational method GiRaF based on a fast biclique enumeration algorithm was applied to identify the sets of taxa with differential phylogenetic placement.

We compared HopPER with the above 6 described state-of-the-art methods by their ability to detect reassortment on real test datasets. Table 3 shows the results of the number of reassortants identified by HopPER and other methods. We set the threshold of 0.5 to classify the reassorted and non-reassorted strains in HopPER. The annotation of real test datasets and corresponding reassortment probabilities can be found in Additional file 1: Table S2. According to the results, our approach easily picked up reassortants where the strains varied in hosts across different periods. Overall, 178 out of 208 strains were successfully detected as reassortants. Looking at outcomes in each dataset, it is apparent that all the similar swine-origin H3N2 influenza strains were recognized as reassortants. Perhaps the number of test genomes on this dataset was not significant and the TPV was only 0.806 on de Silva et al. dataset, slightly worse than other datasets. Some of the reassortant strains identified by Silva et al. were reported for the first time. This could decrease the confidence of the candidates as true reassortant strains. Nevertheless, the evaluation on the real datasets displayed strong evidence for the characterization of reassortment by HopPER, e.g. the validation on Nagarajan et al. dataset achieved TPV of 0.860, which contained larger quantity of genomes with a diversity of strains.

**Table 3:** The results of reassortant strains identified using HopPER that was validated by alternative methods for reassortment analysis

Datasets	Number of genomes	Original Methods	Identified number by HopPER	TPV
Karasin et al.	18	Genetic and phylogenetic analyses with cycle sequencing and amplification by reverse transcription-PCR.	16	0.889
Kingsford et al.	16	Enumerating maximal bicliques with a defined incompatibility graph to detect high-probability inconsistencies between the distributions of trees.	14	0.875
Olsen et al.	6	Phylogenetic analysis by the method of maximum parsimony with bootstrap resampling for the genetic characterization of reassortant H3N2 viruses.	6	1.000
Khiabanian et al.	39	Applying statistical methods such as diversity and entropy measures of each segment and its correlations to investigate reassortment partterns.	33	0.846
de Silva et al.	36	Comprehensive analysis based on neighbourhood of each segment and using only nucleotide distance matrix as input to formulate the phylogeny.	29	0.806
Niranjan et al.	93	Graph-incompatibility based reassortment finder that searches large collections of Markov chain Monte Carlo-sampled trees for groups of incompatible splits using a graph mining technique.	80	0.860

One of the most critical strains A/California/04/2009, as the reference strain for the 2009 pandemic H1N1 virus, was estimated to be reassortant with the probability of 0.885. Of particular interest was the potential host adaptation for individual segments of the genome. Selected avian, human and swine genomic strains are shown in Table 4, indicating the reassortment patterns based on host tropism and reassortment probabilities. The results incorporated the most likely host adaptation for each protein. Most of the reassortants displayed a diversity of host adaptation of influenza sequences in the genome. Table 4 indicates that more than one host species exists in all genomes except the strain A/domestic teal/Hunan/79/2005, which is estimated as a reassortant with the probability of 0.701, with the host tropism for each segment being the same. Another finding was that the reassortment probability of strain A/domestic teal/Hunan/79/2005 was not high compared with others. We may infer that interspecies transmission of influenza viruses had a direct impact on our probability estimation. Correspondingly, we would obtain more credible reassortment events if we can demonstrate that the sequences in the genome stemmed from different species.

**Table 4:** Reassortment patterns on host distribution of selected avian (0), human (1) and swine (2) strains and the gap ’-’ denoted the missing sequence in the genome

	Strain	Subtype	HA	M2	NA	NP	NS1	PA	PB1	PB2	*RE*^*prob*^
Avian	A/domestic teal/Hunan/79/2005	H5N1	0	0	0	0	0	0	0	0	0.701
	A/pekin duck/California/P30/2006	H4N2	0	0	0	0	0	2	0	0	0.856
	A/mallard/Pennsylvania/454069-12/2006	H5N4	0	0	1	0	0	0	0	0	0.804
	A/chicken/Hubei/C1/2007	H9N2	0	2	0	0	2	2	0	0	0.976
Human	A/California/05/2009	H1N1	1	1	1	1	-	1	1	1	0.888
	A/Texas/05/2009	H1N1	1	2	2	1	1	1	1	1	0.993
	A/California/04/2009	H1N1	1	2	1	1	1	1	1	1	0.885
	A/New Jersey/1976	H1N1	2	0	1	1	1	1	1	1	0.984
Swine	A/Thailand/271/2005	H1N1	1	-	1	0	2	2	2	2	0.995
	A/swine/Ontario/00130/97	H3N2	2	1	1	2	2	1	1	2	1
	A/swine/Ontario/53518/03	H1N1	2	2	2	2	2	2	1	2	0.959
	A/swine/Hong Kong/273/1994	H1N1	1	2	1	1	2	2	1	2	0.999

Reassortant strains are implicated in several major pandemics in history with reassortments occurring across different hosts. An example is swine-origin reassortant, which comprises genes derived from avian, human and classical swine [8]. More attention is needed for the reassortant strains when the complement of individual protein sequences are from three or more different host species detected by HopPER. Besides, the emergence of novel HA segment in a reassorted genome is crucial for the outbreak of potential pandemics that has to be considered.

Moreover, we were able to further identify latent breakdowns in the ancestry of known reassortants and give insights for interspecies transmission and evolution of influenza viruses. For example, in A/swine/Ontario/53518/03, we found that the segment PB1 was derived from human influenza virus lineages while all the remaining genes were of classical swine lineage [42]. The H3N2 viruses recovered from Canada in January 1997 like A/swine/Ontario/00130/97 from Ontario isolates, which were regarded as wholly human influenza viruses [40]. It was consistent with our results that four segments M2, NA, PA and PB1 originated from human influenza viruses, suggesting strong interspecies transmission of the different clades. Similarly, the highly pathogenic avian influenza (HPAI) H5N1 lineage in Asia has demonstrated various combinations of its genes to form several generations of multiple reassortants [51]. The precursor of H5N1 strain A/Goose/Guangdong/1/96 and the re-emerging strain A/peregrine falcon/Hong Kong/2142/2008 were reassortants with probabilities 0.748 and 0.546 respectively (Additional file 1: S2). The complex reassortment mechanism and manifold possibility of combination could adversely affect the host tropism prediction and overestimate the probability of reassortment, but HopPER has manifested the robustness of its capability to identify reassortment and also provided perspectives for evolutionary patterns.

### Evaluation on synthetic datasets

To further verify our model’s ability to identify induced reassortants and assess performance in a controlled setting, we carried out experiments on lab-synthesized reassortant strains. These synthetic strains were regarded as the true label on the detection of reassortants. The synthetic dataset was divided into complete and incomplete genomes that contained 85 and 25 samples respectively. According to the rules of our model, the data of incomplete genomes contained two different sequences at least. We have summarized the results of reassortment detection on both complete and incomplete strains by HopPER in Table 5. HopPER correctly identified 19 out of 25 reassortants for incomplete genomes and 83 out of 85 reassortants for complete genomes on synthetic strains. The probabilities of reassortment can be seen in Additional file 1: Table S4. Though the incomplete information of genomes likely influenced the prediction of reassortment, the TPV achieved by HopPER on laboratory dataset (0.927) was more persuasive compared with the real dataset (0.855). On observation, the false positives reported by our model were dominated by incomplete samples. We have found that all these false positives only contain HA and NA proteins while most of the rest of incomplete genomes have more than two different segments (Additional file 1: Table S5). In general, we can infer that the number of available segments in a genome is a critical factor impacting the reliable estimation of reassortment probability. Despite this, the false positive rate was still less than 0.1 on synthetic datasets.

**Table 5:** The number of predicted reassortant strains identified by HopPER for complete and incomplete genomes in both real and synthetic datasets

Genomes	Integrity of genome	Predicted reassortants/total number
Real	Complete	154/173
	Incomplete	24/35
Synthetic	Complete	83/85
	Incomplete	19/25

It is usually hard or impossible to detect the reassortment by either our model or other methods if the input genome is incomplete. It also poses great challenges for any other computational tools to identify reassortment events with lots of missing information in the genome. We are able to explore the reassortant strains in synthetic genomes by estimating the probabilities without constraining the integrity of genomes. Though the reassortment analysis on incomplete genomes brings uncertainty of probability estimation and increases the difficulty of identifying reassortment, the results are not greatly affected using HopPER. We have successfully identified 24 out of 35 and 19 out of 25 incomplete strains in real and synthetic datasets respectively. The TPVs of reassortment detection on incomplete strains has achieved noteworthy performance in comparison to complete ones. However, a look into the unsuccessful cases of incomplete strains finds most of the failures in genomes with only 2 segments. We also list the predicted reassortant strains by the number of available sequences in the genome (Additional file 1: Table S6). It demonstrates the effectiveness of HopPER in predicting reassortment of incomplete strains.

### Analysis on reassortment history

Since the emergence of 1918 Spanish pandemic, influenza A viruses have circulated and caused substantial morbidity and mortality in humans [52]. Despite the long-term existence of the influenza virus, the influence of the reassortment in the expected transmission properties of influenza viruses is still an area of active research. A study on 71 representative complete genomes sampled between 1918 to 2006 showed reassortment occurred frequently throughout the evolutionary history of the virus [53]. Though some reassortment events would not cause severe infections or lead to outbreaks, reassortment still plays an important role in the process of evolution and epidemiology for influenza viruses, particularly when considering transmission from avian or swine host populations into human populations. For example, pigs have been known as a mixing vessel with multiple reassortment events occurring. While most of the cases were mild to humans, three out of four pandemics are related to the reassorted swine strains. It is clear that the reassortment between influenza viruses from different host species can generate novel pandemic-potential strains. These antigenic and genetic novel strains are usually not well matched to the contemporaneous vaccines, and so existing vaccines offer little protection [10]. Detecting reassortment frequency among influenza viruses is also a crucial aspect to capture evolutionary history [54].

**Fig. 3:**
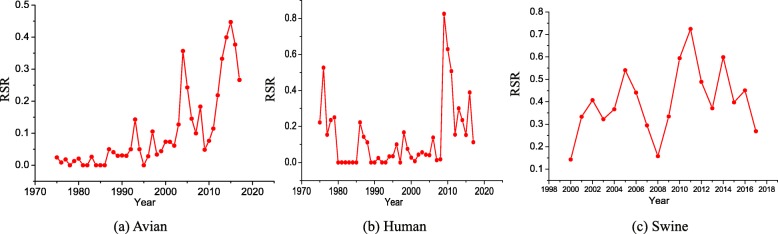
The RSR of three distinct host species of influenza strains across different years detected by HopPER. **a** The RSR of avian species from 1988 to 2017. **b** The RSR of human species from 1975 to 2017. **c** The RSR of swine species from 2000 to 2017

We applied HopPER to investigate the reassortment history on avian, human and swine species respectively. We utilize the RSR to illustrate the variety of reassortant strains. Figure 3 presents the RSR of influenza strains on three distinct species across different years. The experiments are conducted on the years with more than 20 genomes. The results reflect the complex reassortment histories and suggest the reliability compared with the actual evolutionary patterns. In Fig. 3a, the RSR sustains a relative low level until 2004, when the highly pathogenic avian influenza (HPAI) virus of the H5N1 subtype has re-emerged [55]. The HPAI H5N1 virus results from its ability to transmit through both human and bird hosts, leading to novel reassortant strains [56]. The human species describes the different situations in which RSR reaches the local peaks around the pandemic years. The RSR starts to decrease after the outbreak of 1976 pandemic when a new H1N1 strain predominated. After that, another pandemic occurred in 2009 caused by a triple reassortant swine-origin human strain during the time there is a rapid increase of RSR in Fig. 3b. The RSR of swine species varies differently from avian and human and it gains high value, except in 2008. We infer that the swine species, as the mixing vessel, more frequently participates in the reassortment process with both avian and human strains. According to Fig. 3, the RSR remains at relatively high level after 2009. This is because the progeny strains of these 2009 strains are still circulating around the world. Haemagglutination inhibition (HI) tests with post-infection ferret antisera indicates that the majority of A(H1N1)pdm09 viruses are antigenically homogeneous and closely related to the vaccine virus A/California/7/2009 [57]. It is noteworthy as a possible indication for the resurgence of another potential pandemic or epidemic after the current reassortant strains have been in circulation.

## Conclusions

We have developed a novel method HopPER for probability estimation of influenza reassortment based on host prediction. While the development of HopPER mainly focuses on influenza datasets, the model could also be helpful for the research of other viral datasets that contain different host species. We have demonstrated our model by different real and synthetic datasets and validated the results by comparison with alternative methods. HopPER can also be leveraged to detect any known complete or incomplete strains for reassortment identification and reassortant strains with robustness. So it is possible to build an automatic surveillance system to monitor the transmission and reassortment for influenza viruses. We believe this model would facilitate rapid reassortment detection and provide perspectives for the evolutionary patterns of emergent new influenza strains.

## Supplementary information

**Additional file 1** **Table S1** The division of amino acid groups based on physicochemical properties and amino acid indices. **Table S2** The strain names of real datasets and its corresponding reassortment probability estimations in random forest for each genome. **Table S3** The strain names of synthetic datasets and its corresponding reassortment probability estimations in random forest for each strain. (CG: complete genome, IG: incomplete genome) **Table S4** Reassortment patterns of incomplete synthetic strains that ’0’ is avian host, ’1’ is human host, ’2’ is swine host and ’-’ stands for - sequences. **Table S5** The number of predicted reassortant strains identified by HopPER in the case of different number of available sequences contained in the genome. **Table S6** The number of predicted reassortant strains identified by HopPER in the case of different number of available sequences contained in the genome.

## Data Availability

The raw sequences of influenza A viruses are deposited on the National Center for Biotechnology Information (NCBI) influenza virus resource and Global Initiative on Sharing All Influenza Data (GISAID). The accession information of strains validated for the proposed model can be found at: https://drive.google.com/open?id=1mP77pW6icokhUVGm8WnWWIvoF0r8Af1c.

## Abbreviations

HopPER: Host-prediction-based Probability Estimation of Reassortment
HA: hemagglutinin
NA: neuraminidase
PA: polymerase acidic protein
PB1: polymerase basic protein 1
PB2: polymerase basic protein 2
NP: nucleoprotein
NS1: non-structural protein 1
NS2: non-structural protein 2
PB1-F2: polymerase basic accessory protein
PA-X: polymerase acidic protein X
GiRaF: Graph-incompatibility-based Reassortment Finder
CTD: Composition, Transition and Distribution
NCBI: National Center for Biotechnology Information
GISAID: Global Initiative on Sharing All Influenza Data
G-means: geometric means
MCC: Matthew’s correlation coefficient
TPV: true positive value
S-OIV: swine-origin influenza viruses
HPAI: highly pathogenic avian influenza
HI: Haemagglutination inhibition
