# Supplementary material for: HopPER: an adaptive model for probability estimation of influenza reassortment through host prediction
Source: BMC Med Genomics. 2020 Jan 23;13:9. doi: 10.1186/s12920-019-0656-7 (PMC6979075; doi:10.1186/s12920-019-0656-7)
Supplement: Supplementary file 1 — Additional file 1 Table S1 The division of amino acid groups based on physicochemical properties and amino acid indices. Table S2 The strain names of real datasets and its corresponding reassortment probability estimations in random forest for each genome. Table S3 The strain names of synthetic datasets and its corresponding reassortment probability estimations in random forest for each strain. (CG: complete genome, IG: incomplete genome) Table S4 Reassortment patterns of incomplete synthetic strains that ’0’ is avian host, ’1’ is human host, ’2’ is swine host and ’-’ stands for - sequences. Table S5 The number of predicted reassortant strains identified by HopPER in the case of different number of available sequences contained in the genome. Table S6 The number of predicted reassortant strains identified by HopPER in the case of different number of available sequences contained in the genome. [file 12920_2019_656_MOESM1_ESM.pdf]

Table S1. The division of amino acid groups based on physicochemical properties and amino acid indices.

Attributes	Group 1	Group 2	Group 3
Hydrophobicity	Polar<br><br>Q, E, R, K, D, N	Neutral<br><br>G, P, H, A, S, T, Y	Hydrophobic<br><br>C, V, F, L, I, M, W
Polarizability	0-1.08<br><br>S, D, G, A, T	0.128-0.186<br><br>C, Q, I, P, N, V, E, L	0.219- 0.409<br><br>Y, M, K, R, H, F, W
Normalized Van der Waals	0-2.78<br><br>S, C, G, A, T, P, D	2.95-4.0<br><br>E, Q, N, V, I, L	4.0-8.1<br><br>K, F, M, H, R, Y, W
Polarity	4.9-6.2<br><br>W, C, L, I, F, M, V, Y	8.0-9.2<br><br>T, G, P, A, S	10.4-13.0<br><br>K, N, H, Q, R, E, D
Solvent Accessibility	Buried<br><br>A, I, F, C, G, L, V, W	Exposed<br><br>R, K, Q, E, N, D	Intermediate<br><br>M, S, P, T, H, Y
Secondary Structure	Helix<br><br>E, A, L, M, Q, K, R, H	Strand<br><br>V, I, Y, C, W, F, T	Coil<br><br>G, N, P, S, D
Charge	Positive<br><br>K, R	Neutral<br><br>A, N, C, Q, G, H, I, L, M, F, P, S, T, W, Y, V	Negative<br><br>D, E

Table S2. The strain names of real datasets and its corresponding reassortment probability estimations in random forest for each genome.

Datasets	Strain name	Reassortment probability
Karasin et al.	A/Swine/Minnesota/593/99	0.748
	A/Swine/Iowa/533/99	0.753232328
	A/Swine/Iowa/569/99	0.74368
	A/swine/Ontario/00130/97	1
	A/Swine/Nebraska/209/98	0.7997
	A/Swine/Illinois/100085A/01	0.37
	A/Swine/North Carolina/98225/01	0.695872
	A/Swine/Illinois/100084/01	0.58
	A/Swine/Ohio/891/01	0.4456
	A/Swine/Indiana/P12439/00	0.8272
	A/Swine/North Carolina/93523/01	0.5816
	A/Swine/Minnesota/55551/00	0.857009
	A/Swine/Iowa/930/01	0.964
	A/swine/Ontario/53518/03	0.9595
	A/swine/Ontario/23866/04	0.8803
	A/swine/Ontario/48235/04	0.981629
	A/swine/Ontario/55383/04	0.814931
	A/swine/Ontario/11112/04	0.7732
Kingsford et al.	A/swine/Ratchaburi/NIAH550/2003	0.8704
	A/swine/Chonburi/05CB1/2005	0.1
	A/swine/Ratchaburi/NIAH1481/2000	0.271
	A/Thailand/271/2005	0.995392
	A/Texas/05/2009	0.972632
	A/swine/Ratchaburi/NIAH874/2005	0.5383
	A/swine/Chonburi/06CB2/2006	0.64
	A/swine/Nakhonpathom/NIAH5861/2005	0.9496
	A/swine/Ratchaburi/NIAH59/2004	0.782272
	A/California/05/2009	0.816211
	A/swine/Chonburi/NIAH977/2004	0.6472
	A/swine/Chonburi/NIAH9469/2004	0.78832
	A/swine/Chachoengsao/2003	0.524
	A/swine/Chonburi/NIAH589/2005	0.8056
	A/swine/Saraburi/NIAH13021/2005	0.7984
	A/swine/Thailand/HF6/2005	0.64
Olsen et al.	A/swine/Manitoba/12707/2005	0.768533
	A/Ontario/RV1273/2005	0.80656
	A/swine/BritishColumbia/28103/2005	0.768533
	A/swine/Alberta/14722/2005	0.76564
	A/turkey/Ontario/31232/2005	0.82
	A/swine/Ontario/33853/2005	0.80656
Khiabanian et al.	A/swine/Spain/33601/2001	0.862
	A/Swine/Indiana/9K035/99	0.8056

	A/swine/Shanghai/1/2007	0.59218
	A/swine/Cloppenburg/IDT4777/2005	0.37
	A/swine/Ohio/24366/07	0.857174
	A/swine/Alberta/14722/2005	0.819004
	A/swine/Ontario/53518/03	0.838
	A/swine/Ontario/57561/03	0.75424
	A/swine/Tennessee/82/1977	0.591207
	A/Swine/North Carolina/35922/98	0.999611
	A/swine/Tennessee/48/1977	0.424
	A/swine/Ontario/6/1981	0.582287
	A/swine/Tennessee/96/1977	0.63904
	A/swine/Korea/CY04/2007	0.934682
	A/Swine/Nebraska/209/98	0.80257
	A/swine/Tennessee/23/1976	0.5455
	A/swine/Tennessee/15/1976	0.72321
	A/Swine/Minnesota/55551/00	0.666355
	A/swine/Korea/CY07/2007	0.958
	A/swine/Tennessee/62/1977	0.5488
	A/swine/Tennessee/61/1977	0.52
	A/swine/Ontario/48235/04	0.969382
	A/swine/Zhejiang/1/2004	0.371636
	A/swine/Tennessee/64/1977	0.568225
	A/swine/Korea/CAN01/2004	0.55
	A/swine/Miyazaki/1/2006	0.799962
	A/swine/Alberta/56626/03	0.806208
	A/swine/North Carolina/2003	0.962242
	A/swine/Iowa/1/1976	0.67276
	A/swine/Spain/53207/2004	0.1
	A/swine/MI/PU243/04	0.588945
	A/swine/Italy/1521/98	0.99784
	A/swine/Missouri/2124514/2006	1
	A/swine/Wisconsin/1915/1988	0.510112
	A/swine/MI/PU243/04	0.588945
	A/swine/Minnesota/5892-7/1979	0.624356
	A/swine/Iowa/1/1986 1986//	0.28558
	A/swine/Tennessee/19/1976	0.278222
	A/swine/Ontario/11112/04	0.74485
de Silva et al.	A/equine/Lexington/1/1966	0.223614
	A/turkey/Ontario/6213/1966 1966/06/11	0.541002
	A/turkey/Wisconsin/1/1968 1968/06/11	0.984254
	A/turkey/MO/21939/1987	0.655285
	A/duck/Shimane/188/1999	0.97638
	A/mallard/Netherlands/02/2000	0.616691
	A/environment/Hong Kong/674.15/2002	0.137349
	A/chicken/Henan/01/2004	0.76209
	A/domestic green-winged teal/Hunan/79/2005	1

	A/teal/Italy/3931-38/2005	0.223614
	A/common murre/Oregon/19497-004/2005	0.80398
	A/whooper swan/Mongolia/232/2005 2005/08/09	0.28
	A/mallard/Netherlands/30/2006	0.634458
	A/pekin duck/California/P30/2006 2006/03/06	0.992941
	A/mallard/Pennsylvania/454069-12/2006	1
	A/northern shoveler/Netherlands/1/2006	0.533615
	A/chicken/Laos/P0130/2007	0.691544
	A/environment/Hunan/5-32/2007	0.963261
	A/little egret/Hong Kong/8550/2007	0.201397
	A/chicken/Kobe/aq26/2001	0.997519
	A/chicken/Hubei/C1/2007	0.999395
	A/mallard/Maryland/798/2007	1
	A/Eurasian wigeon/Netherlands/3/2007	0.701387
	A/mallard/Netherlands/17/2007	0.5464
	A/environment/Hunan/6-69/2008	0.098338
	A/peregrine falcon/Hong Kong/2142/2008	0.9352
	A/northern shoveler/Interior Alaska/8BM3470/2008	0.529021
	A/goose/Czech Republic/1848-K9/2009	0.1
	A/swine/Guangdong/102/2002	0.856
	A/swine/Guangxi/wz/2004	0.979895
	A/swine/Guangdong/wxl/2004	0.668386
	A/swine/Shandong/3/2005	0.748011
	A/swine/Shandong/1123/2008	0.989112
	A/New Jersey/1976	0.593842
	A/canine/Korea/1/2010	0.776009
	A/Goose/Guangdong/1/96	0.5375
Niranjan et al.	A/swine/Chonburi/NIAH977/2004	0.6472
	A/swine/Wisconsin/11/1980	0.629119
	A/swine/Belgium/WVL1/1979	0.999883
	A/swine/OH/511445/2007	0.52677
	A/swine/Chonburi/NIAH9469/2004	0.78832
	A/swine/Ontario/1/1981	0.627292
	A/swine/Tennessee/9/1978	0.67114
	A/swine/Niigata/1/1977	0.668669
	A/swine/Wisconsin/8/1980	0.53716
	A/swine/Minnesota/5892-7/1979	0.624356
	A/swine/Tennessee/8/1978	0.584896
	A/California/04/2009 2009/04/01	0.88453
	A/swine/Ratchaburi/NIAH1481/2000	0.271
	A/swine/Iowa/4/1976	0.67276
	A/swine/Wisconsin/629/1980	0.74016
	A/swine/Hokkaido/2/1981	0.83872
	A/swine/Tennessee/49/1977	0.595

	A/swine/Ontario/53518/03	0.838
	A/swine/Kansas/3024/1987	0.69712
	A/swine/Tennessee/79/1977	0.75255
	A/swine/Iowa/17672/1988	0.992895
	A/swine/Tennessee/10/1978	0.479305
	A/swine/Tennessee/65/1977	0.568225
	A/swine/Ontario/4/1981	0.707601
	A/swine/Iowa/2/1985	0.232638
	A/swine/Maryland/23239/1991	0.863427
	A/swine/Alberta/56626/03	0.806208
	A/swine/Iowa/1/1985	0.232638
	A/swine/Wisconsin/661/1980	0.566933
	A/swine/Chonburi/NIAH589/2005	0.776
	A/California/06/2009 2009/04/16	0.887997
	A/swine/Ontario/57561/03	0.75424
	A/swine/Tennessee/7/1978	0.5455
	A/swine/California/T9001707/1991	0.973049
	A/swine/Shanghai/1/2005	0.568
	A/swine/Nebraska/123/1977	0.81856
	A/swine/Iowa/24297/1991	0.941085
	A/swine/Tennessee/62/1977	0.5488
	A/swine/Tennessee/61/1977	0.52
	A/swine/Wisconsin/641/1980	0.566933
	A/swine/Ontario/11112/04	0.74485
	A/swine/Ratchaburi/NIAH550/2003	0.8704
	A/swine/Memphis/1/1990	0.975226
	A/swine/France/WVL8/1992	0.998236
	A/swine/Tennessee/64/1977	0.568225
	A/swine/Korea/CAN01/2004	0.55
	A/swine/Ontario/2/1981	0.66528
	A/swine/Kyoto/3/1979	0.929316
	A/swine/Chachoengsao/NIAH587/2005	0.776
	A/swine/Wisconsin/663/1980	0.695987
	A/swine/Iowa/31483/1988	0.958463
	A/swine/Tennessee/25/1977	0.4924
	A/swine/Hong Kong/273/1994	0.999043
	A/swine/Ontario/3/1981	0.659813
	A/swine/Ontario/23866/04	0.8803
	A/swine/Kansas/3228/1987	0.69712
	A/swine/Ohio/24366/07	0.857174
	A/swine/Iowa/1/1987	0.663832
	A/swine/Korea/CAS08/2005	0.625222
	A/swine/Iowa/3/1985	0.232638
	A/swine/Tennessee/10/1977	0.535
	A/swine/Iowa/2/1987	0.244
	A/swine/Shanghai/1/2005	0.3

	A/swine/Wisconsin/1915/1988	0.510112
	A/swine/Wisconsin/30747/1976	0.84
	A/swine/Tennessee/11/1978	0.679305
	A/swine/Kentucky/1/1976	0.8485
	A/swine/Wisconsin/30954/1976	0.673975
	A/swine/Iowa/1/1986	0.28558
	A/swine/Tennessee/3/1978	0.78587
	A/swine/Ontario/6/1981	0.582287
	A/swine/Ontario/7/1981	0.66528
	A/chicken/Jilin/hm/2003	0.7975
	A/duck/Yokohama/aq10/2003	0.383026151
	A/Ck/HK/37.4/2002	0.655
	A/swine/Anhui/cb/2004	0.99118
	A/golden mountain thrush/Fujian/376/04	0.974863
	A/chicken/Jilin/hn/2003	0.76
	A/turkey/Turkey/1/2005	0.709696
	A/chicken/Hebei/718/2001	0.1
	A/chicken/Jilin/ho/2003	0.784
	A/chicken/Jilin/hl/2004	0.405296154
	A/Muscovy duck/Vietnam/48/2007	0.91810346
	A/Swine/Fujian/F1/2001	1
	A/magpie robin/Hong Kong/1897/2008	0.597965702
	A/duck/Vietnam/210/2005	0.75498836
	A/Chicken/Hong Kong/858.3/01	0.99955
	A/chicken/Primorje/1/2008	0.53577998
	A/chicken/Hunan/1793/2007	0.61444
	A/peregrine falcon/Hong Kong/2142/2008	0.5464
	A/goose/Shantou/3624/2006	0.904
	A/chicken/Krasnodar/300/07	0.285714286
	A/Cygnus olor/Germany/R1372/2007	0.847855

Table S3. The strain names of synthetic datasets and its corresponding reassortment probability estimations in random forest for each strain. (CG: complete genome, IG: incomplete genome)

Type	Strain name	Reassortment probability
CG	A/reassortant/NYMC X-175A(Uruguay/716/2007 x Puerto Rico/8/1934)	0.7084
	A/reassortant/NYMC X-173A(NYMC X-157 x South Dakota/06/2007)	0.7813
	A/reassortant/X-53(Puerto Rico/8/1934 x New Jersey/11/1976)	0.994751
	A/reassortant/NYMC X-225A(A/Puerto Rico/8/1934 x A/Hawaii/22/2012)	0.76672
	A/reassortant/NYMC X-151(Fujian/445/2003 x Puerto Rico/8/1934)	0.8704
	A/reassortant/X-61(Texas/1/1977 x Puerto Rico/8/1934)	0.850701
	A/reassortant/X-139(X-31B x New Caledonia/20/1999)	0.79588
	A/reassortant/NYMC X-207(Perth/10/2010 x Puerto Rico/8/1934)	0.902124
	A/reassortant/X-37(England/42/1972 x Puerto Rico/8/1934)	0.951011
	A/reassortant/X-119(Puerto Rico/8/1934 x Harbin/15/1992)	0.381084
	A/reassortant/NYMC X-211(NYMC X-157 x St. Petersburg/100/2011)	0.908917
	A/reassortant/NYMC X-165(Nepal/921/2006 x Puerto Rico/8/1934)	0.874
	A/reassortant/NYMC X-167C(Wisconsin/03/2007 x Puerto Rico/8/1934)	0.728434
	A/reassortant/NYMC X-177(Hong Kong/1870/2008 x Puerto Rico/8/1934)	0.73756
	A/reassortant/NYMC X-217(A/Puerto Rico/8/1934 x A/Victoria/361/2011)	0.79588
	A/reassortant/X-123A(Johannesburg/33/1994 x Puerto Rico/8/1934)	0.884264
	A/reassortant/NYMC X-211B(NYMC X-157 x St. Petersburg/100/2011)	0.946883
	A/reassortant/NYMC X-193A(Finland/97/2009 x Puerto Rico/8/1934)	0.8542
	A/reassortant/NYMC X-197(Brisbane/11/2010 x Puerto Rico/8/1934)	0.996548
	A/reassortant/NYMC X-203(A/Puerto Rico/8/1934 x A/Minnesota/11/2010)	0.853034
	A/reassortant/NYMC X-199(Rhode Island/01/2010 x Puerto Rico/8/1934)	0.891249
	A/reassortant/NYMC X-177B(Hong Kong/1870/2008 x Puerto Rico/8/1934)	0.76672
	A/reassortant/X-143(Ulan Ude/01/2000 x Puerto Rico/8/1934)	0.877528
	A/reassortant/NYMC X-183(Wisconsin/15/2009 x Puerto Rico/8/1934)	0.8704
	A/reassortant/X-175C(Uruguay/716/2007 x Puerto Rico/8/1934)	0.8542
	A/reassortant/X-137(Moscow/10/1999 x Puerto Rico/8/1934)	0.908146
	A/reassortant/NYMC X-167A(Wisconsin/03/2007 x Puerto Rico/8/1934)	0.780032
	A/reassortant/NYMC X-147(Wyoming/03/2003 x Puerto Rico/8/1934)	0.816292
	A/reassortant/X-129(South Africa/1147/1996 x Puerto Rico/8/1934)	0.763804
	A/reassortant/NYMC X-213(A/Puerto Rico/8/1934 x A/Indiana/10/2011)	1
	A/reassortant/X-121(Shangdong/9/1993 x Puerto Rico/8/1934)	0.79588
	A/reassortant/NYMC X-189(Hong Kong/26560/2009 x Puerto Rico/8/1934)	0.82504
	A/reassortant/NYMC X-187A(Victoria/210/2009 x Puerto Rico/8/1934)	0.834663
	A/reassortant/NYMC X-153A(Texas/40/2003 x Puerto Rico/8/1934)	0.73756
	A/reassortant/NYMC X-205(NYMC X-157 x South Carolina/10/2010)	0.884446
	A/reassortant/NYMC X-223(A/Puerto Rico/8/1934 x A/Texas/50/2012)	0.763804
	A/reassortant/NYMC X-167B(Wisconsin/03/2007 x Puerto Rico/8/1934)	0.613225
	A/reassortant/NYMC X-181A(A/NYMC X-157 x California/07/2009)	0.851314
	A/reassortant/X-41(Port Chalmers/1/1973 x Puerto Rico/8/1934)	0.84448
	A/reassortant/H9N2:pH1N1_RGpassage7(quail/Hong Kong/G1/1997 x California/04/2009)	0.995587
	A/reassortant/NYMC X-169(Brisbane/09/2006 x Puerto Rico/8/1934)	0.853034

	A/reassortant/NYMC X-167(Wisconsin/03/2007 x Puerto Rico/8/1934)	0.7084
	A/reassortant/IVR-148(Brisbane/59/2007 x Texas/1/1977)	0.836704
	A/reassortant/X-109(Beijing/353/1989 x Puerto Rico/8/1934)	0.79588
	A/reassortant/NYMC X-155(Wellington/01/2004 x Puerto Rico/8/1934)	0.750682
	A/reassortant/NYMC X-179A(NYMC X-157 x A/California/07/2009)	0.851314
	A/reassortant/NYMC X-203A(A/Puerto Rico/8/1934 x A/Minnesota/11/2010)	0.853034
	A/reassortant/X-79(Philippines/2/1982 x Puerto Rico/8/1934)	0.7084
	A/reassortant/NYMC X-173B(NYMC X-157 x South Dakota/06/2007)	0.73756
	A/reassortant/NYMC X-181(A/NYMC X-157 x California/07/2009)	0.851314
	A/reassortant/NYMC X-169A(Brisbane/09/2006 x Puerto Rico/8/1934)	0.7084
	A/reassortant/X-141(Panama/2007/1999 x Puerto Rico/8/1934)	0.73756
	A/reassortant/NYMC X-211A(NYMC X-157 x St. Petersburg/100/2011)	0.84787
	A/reassortant/NYMC X-187(Victoria/210/2009 x Puerto Rico/8/1934)	0.834663
	A/reassortant/NYMC X-225(A/Puerto Rico/8/1934 x A/Hawaii/22/2012)	0.79588
	A/reassortant/NYMC X-195A(Finland/97/2009 x Puerto Rico/8/1934)	0.728434
	A/reassortant/X-97(Sichuan/2/1987 x Puerto Rico/8/1934)	0.906688
	A/reassortant/X-121(Puerto Rico/8/1934 x Shangdong/9/1993)	0.79588
	A/reassortant/IgYRP2(California/07/2004 x Puerto Rico/8/1934)	0.908875
	A/reassortant/X-37a(England/42-MRC-2/1972 x Puerto Rico/8/1934)	0.98367
	A/reassortant/X-7F1(X-7 x Rockefeller Institute/5/1957)	0.7732
	A/reassortant/X-83(Chile/1/1983 x X-31)	0.853034
	A/reassortant/NYMC X-191(Philippines/219/2009 x Puerto Rico/8/1934)	0.9712
	A/reassortant/NYMC X-173C(NYMC X-157 x South Dakota/06/2007)	0.73756
	A/reassortant/NYMC X-177A(Hong Kong/1870/2008 x Puerto Rico/8/1934)	0.82504
	A/reassortant/X-7(NWS/1934 x Rockefeller Institute/5/1957)	1
	A/reassortant/NYMC X-173(NYMC X-157 x South Dakota/06/2007)	0.73756
	A/reassortant/NYMC X-185xp(Guangdong-Luohu/1256/2009 x Puerto Rico/8/1934)	0.73756
	A/reassortant/X-145(California/32/1999 x Puerto Rico/8/1934)	0.455143
	A/reassortant/NYMC X-221(A/Puerto Rico/8/1934 x A/Ohio/2/2012)	0.7084
	A/reassortant/X-99(Shanghai/11/1987 x Puerto Rico/8/1934)	0.853034
	A/reassortant/NYMC X-215(A/Puerto Rico/8/1934 x A/Brisbane/299/2011)	0.73756
	A/reassortant/NYMC X-185(Guangdong-Luohu/1256/2009 x Puerto Rico/8/1934)	0.982504
	A/reassortant/NYMC X-223A(A/Puerto Rico/8/1934 x A/Texas/50/2012)	0.763804
	A/reassortant/NYMC X-227(A/Puerto Rico/8/1934 x A/Indiana/21/2012)	1
	A/reassortant/NYMC X-179(NYMC X-157 x A/California/07/2009)	0.970263
	A/reassortant/NYMC X-193(Finland/97/2009 x Puerto Rico/8/1934)	0.836704
	A/reassortant/NYMC X-205A(NYMC X-157 x South Carolina/10/2010)	0.810706
	A/reassortant/NYMC X-207A(Perth/10/2010 x Puerto Rico/8/1934)	0.750057
	A/reassortant/CDC2005712034(California/07/2004 x Puerto Rico/8/1934)	0.93925
	A/reassortant/NYMC X-157 CL-3(New York/55/2004 x Puerto Rico/8/1934)	0.73756
	A/reassortant/X-47(Victoria/3/1975 x Puerto Rico/8/1934)	0.836704
	A/reassortant/X-65(USSR/90/1977 x Puerto Rico/8/1934)	0.851197
	A/reassortant/X-91(Leningrad/360/1986 x Puerto Rico/8/1934)	0.947512
	A/reassortant/X-73(Bangkok/1/1979 x Puerto Rico/8/1934)	0.869363
	A/reassortant/NYMC X-217A(A/Puerto Rico/8/1934 x A/Victoria/361/2011)	0.959176
	A/reassortant/NYMC X-181B(A/NYMC X-157 x California/07/2009)	0.866183

IG	A/reassortant/X157(New York/55/2004 x Puerto Rico/8/1934)	0.9925
	A/reassortant/NYMC X-185	0.64
	A/reassortant/NYMC X-197	0.6
	A/reassortant/IVR-148(Brisbane/59/2007 x Texas/1/1977)	0.76672
	A/reassortant/CBER_RG1(duck/Laos/3295/2006 x Puerto Rico/8/1934)	0.62
	A/reassortant/IDCDC-RG18(Texas/05/2009 x New York/18/2009 x Puerto Rico/8/1934)	0.264775
	A/reassortant/IgYRP16(California/07/2004 x Puerto Rico/8/1934)	0.9271
	A/REASSORTANT/IDCDC-RG56B(HONG KONG/125/2017 X PUERTO RICO/8/1934)	0.473333
	A/reassortant/IDCDC-RG20(Texas/05/2009 x Puerto Rico/8/1934)	0.218014
	A/reassortant/NYMC X-183	0.856
	A/reassortant/IDCDC-RG56N(Hong Kong/125/2017 X Puerto Rico/8/1934)	0.473333
	A/reassortant/IDCDC-RG13(Egypt/3300-NAMRU3/2008 x Puerto Rico/8/1934)	0.62
	A/reassortant/RESVIR9(Nanchang/933/1995 x Puerto Rico/8/1934)	0.9949
	A/reassortant/X-99	0.836704
	A/reassortant/X161(Wisconsin/67/2005 x Puerto Rico/8/1934)	0.996976
	A/reassortant/RESVIR17(Panama/2007/1999 x Puerto Rico/8/1934)	0.9942
	A/reassortant/IVR147(Brisbane/10/2007 x Puerto Rico/8/1934)	0.999496
	A/reassortant/JLUMV_RG1(chicken/Egypt/VSVRI/2009 x Puerto Rico/8/1934)	0.5
	A/reassortant/IDCDC-RG42A(Sichuan/26221/2014 X Puerto Rico/8/1934)	0.6
	A/reassortant/NIBRG268(Anhui/1/2013 x Puerto Rico/8/1934)	0.402063
	A/reassortant/X-73	0.836704
	A/reassortant/IDCDC-RG56B	0.473333
	A/reassortant/NYMC X-191	0.68
	A/Philippines/2/82/BS [A/PR/8/24 X A/Phil/2/82 reassortant]	0.6
	A/reassortant/X147(Wyoming/3/2003 x Puerto Rico/8/1934)	0.99064

Table S4. Reassortment patterns of incomplete synthetic strains that ‘0’ is avian host, ‘1’ is human host, ‘2’ is swine host and ‘-‘ stands for - sequences.

Index	Strain name
0	A/reassortant/X157(New York/55/2004 x Puerto Rico/8/1934)
1	A/reassortant/NYMC X-185
2	A/reassortant/NYMC X-197
3	A/reassortant/IVR-148(Brisbane/59/2007 x Texas/1/1977)
4	A/reassortant/CBER_RG1(duck/Laos/3295/2006 x Puerto Rico/8/1934)
5	A/reassortant/IDCDC-RG18(Texas/05/2009 x New York/18/2009 x Puerto Rico/8/1934)
6	A/reassortant/IgYRP16(California/07/2004 x Puerto Rico/8/1934)
7	A/REASSORTANT/IDCDC-RG56B(HONG KONG/125/2017 X PUERTO RICO/8/1934)
8	A/reassortant/IDCDC-RG20(Texas/05/2009 x Puerto Rico/8/1934)
9	A/reassortant/NYMC X-183
10	A/reassortant/IDCDC-RG56N(Hong Kong/125/2017 X Puerto Rico/8/1934)
11	A/reassortant/IDCDC-RG13(Egypt/3300-NAMRU3/2008 x Puerto Rico/8/1934)
12	A/reassortant/RESVIR9(Nanchang/933/1995 x Puerto Rico/8/1934)
13	A/reassortant/X-99
14	A/reassortant/X161(Wisconsin/67/2005 x Puerto Rico/8/1934)
15	A/reassortant/RESVIR17(Panama/2007/1999 x Puerto Rico/8/1934)
16	A/reassortant/IVR147(Brisbane/10/2007 x Puerto Rico/8/1934)
17	A/reassortant/JLUMV_RG1(chicken/Egypt/VSVRI/2009 x Puerto Rico/8/1934)
18	A/reassortant/IDCDC-RG42A(Sichuan/26221/2014 X Puerto Rico/8/1934)
19	A/reassortant/NIBRG268(Anhui/1/2013 x Puerto Rico/8/1934)
20	A/reassortant/X-73
21	A/reassortant/IDCDC-RG56B
22	A/reassortant/NYMC X-191
23	A/Philippines/2/82/BS [A/PR/8/24 X A/Phil/2/82 reassortant]
24	A/reassortant/X147(Wyoming/3/2003 x Puerto Rico/8/1934)

Index	HA	M2	NA	NP	NS1	PA	PB1	PB2
0	-	0	-	0	0	2	0	0
1	-	0	-	1	1	-	-	-
2	-	0	-	-	1	-	-	-
3	1	0	1	1	-	1	1	1
4	0	-	1	-	-	-	-	-
5	1	-	1	-	-	-	-	-
6	1	1	1	-	0	1	0	0
7	0	-	0	-	-	-	-	-
8	1	-	1	-	-	-	-	-
9	1	0	1	1	1	1	-	1
10	0	-	0	-	-	-	-	-
11	0	-	1	-	-	-	-	-
12	-	1	-	2	0	0	0	0
13	1	0	1	1	1	1	-	1

14	-	2	-	0	1	2	0	0
15	-	2	-	0	1	0	1	0
16	-	1	-	2	0	2	1	2
17	0	-	0	-	-	-	-	-
18	1	-	0	-	-	-	-	-
19	0	-	0	-	-	-	-	-
20	1	0	1	1	1	1	-	1
21	0	-	0	-	-	-	-	-
22	1	0	1	1	1	-	-	-
23	-	0	-	-	1	-	-	-
24	-	2	-	2	0	0	0	2

Table S5. The number of predicted reassortant strains identified by HopPER in the case of different number of available sequences contained in the genome.

Available sequence of a genome	Number of test strains (real and synthetic datasets)	Number of reassrotant identified by HopPER
2	14	6
3	4	4
4	6	4
5	8	7
6	15	12
7	13	10
8	258	237

Table S6: The accession information of strains validated for the proposed model

Karasin et al. dataset

ncbiId:AAG01753	ncbiId:AAL87876	ncbiId:AAL87936	ncbiId:ABB86908
ncbiId:AAG01751	ncbiId:AAL87884	ncbiId:AAL87896	ncbiId:ABB86906
ncbiId:AAG01752	ncbiId:AAL87904	ncbiId:AAL87928	ncbiId:ABB86907
ncbiId:AAG01754	ncbiId:AAL87903	ncbiId:AAL87911	ncbiId:ABB86909
ncbiId:AAG01755	ncbiId:AAL87868	ncbiId:AAL87895	ncbiId:ABB86902
ncbiId:AAG01756	ncbiId:AAL87932	ncbiId:AAL87910	ncbiId:ABB86942
ncbiId:AAG01757	ncbiId:AAL87878	ncbiId:AAL87909	ncbiId:ABB86931
ncbiId:AAG01749	ncbiId:AAL87934	ncbiId:AAL87919	ncbiId:ABB86949
ncbiId:AAG01750	ncbiId:AAL87908	ncbiId:AAL87887	ncbiId:ABB86948
ncbiId:AAG01766	ncbiId:AAL87918	ncbiId:AAL87935	ncbiId:ABB86941
ncbiId:AAG01758	ncbiId:AAL87886	ncbiId:AAL87871	ncbiId:ABB86943
ncbiId:AAG01759	ncbiId:AAL87894	ncbiId:AAL87927	ncbiId:ABB86940
ncbiId:AAG01761	ncbiId:AAL87926	ncbiId:AAL87879	ncbiId:ABB86947
ncbiId:AAG01762	ncbiId:AAL87870	ncbiId:AAL87925	ncbiId:ABB86946
ncbiId:AAG01763	ncbiId:AAL87907	ncbiId:AAL87933	ncbiId:ABB86945
ncbiId:AAG01764	ncbiId:AAL87881	ncbiId:AAL87917	ncbiId:ABB86944
ncbiId:AAG01765	ncbiId:AAL87913	ncbiId:AAL87885	ncbiId:ABB86930
ncbiId:AAG01760	ncbiId:AAL87889	ncbiId:AAL87893	ncbiId:ABB86932
ncbiId:AAG01775	ncbiId:AAL87873	ncbiId:AAL87906	ncbiId:ABB86934
ncbiId:AAG01774	ncbiId:AAL87921	ncbiId:AAL87905	ncbiId:ABB86935
ncbiId:AAG01769	ncbiId:AAL87898	ncbiId:AAL87869	ncbiId:ABB86936
ncbiId:AAG01770	ncbiId:AAL87897	ncbiId:AAL87877	ncbiId:ABB86939
ncbiId:AAG01771	ncbiId:AAL87929	ncbiId:ABB86920	ncbiId:ABB86938
ncbiId:AAG01772	ncbiId:AAL87865	ncbiId:ABB86921	ncbiId:ABB86933
ncbiId:AAG01768	ncbiId:AAL87891	ncbiId:ABB86922	ncbiId:ABB86937
ncbiId:AAG01773	ncbiId:AAL87923	ncbiId:ABB86924	
ncbiId:AAG01767	ncbiId:AAL87931	ncbiId:ABB86923	
ncbiId:AAG01784	ncbiId:AAL87902	ncbiId:ABB86925	
ncbiId:AAG01777	ncbiId:AAL87901	ncbiId:ABB86928	
ncbiId:AAG01778	ncbiId:AAL87867	ncbiId:ABB86929	
ncbiId:AAG01776	ncbiId:AAL87875	ncbiId:ABB86927	
ncbiId:AAG01779	ncbiId:AAL87915	ncbiId:ABB86926	
ncbiId:AAG01780	ncbiId:AAL87883	ncbiId:ABB86895	
ncbiId:AAG01781	ncbiId:AAL87890	ncbiId:ABB86891	
ncbiId:AAG01782	ncbiId:AAL87922	ncbiId:ABB86892	
ncbiId:AAG01783	ncbiId:AAL87930	ncbiId:ABB86893	
ncbiId:AAG01787	ncbiId:AAL87900	ncbiId:ABB86897	
ncbiId:AAG01788	ncbiId:AAL87866	ncbiId:ABB86896	
ncbiId:AAG01791	ncbiId:AAL87874	ncbiId:ABB86898	
ncbiId:AAG01792	ncbiId:AAL87899	ncbiId:ABB86899	
ncbiId:AAG01793	ncbiId:AAL87914	ncbiId:ABB86894	
ncbiId:AAG01785	ncbiId:AAL87882	ncbiId:ABB86890	
ncbiId:AAG01786	ncbiId:AAL87880	ncbiId:ABB86904	
ncbiId:AAG01789	ncbiId:AAL87912	ncbiId:ABB86900	
ncbiId:AAG01790	ncbiId:AAL87888	ncbiId:ABB86901	
ncbiId:AAL87916	ncbiId:AAL87872	ncbiId:ABB86903	
ncbiId:AAL87924	ncbiId:AAL87920	ncbiId:ABB86905	

Kingsford et al. dataset

ncbiId:ACP41926	ncbiId:BAH02033	ncbiId:ABY40396	ncbiId:BAH02102
ncbiId:ACP41927	ncbiId:BAH02034	ncbiId:ADG27489	ncbiId:BAH02101
ncbiId:ACP41930	ncbiId:BAH02028	ncbiId:ABY40409	ncbiId:BAH02098
ncbiId:ACP41932	ncbiId:BAH02029	ncbiId:ABY40397	ncbiId:BAH02099
ncbiId:ACP41933	ncbiId:BAH02030	ncbiId:ACM80370	ncbiId:BAH02105
ncbiId:ACP41928	ncbiId:BAH02037	ncbiId:ACM80371	ncbiId:BAH02127
ncbiId:ACP41931	ncbiId:BAH02038	ncbiId:ADG27488	ncbiId:BAH02128
ncbiId:ACP41929	ncbiId:BAH02039	ncbiId:ABY40410	ncbiId:BAH02129
ncbiId:ACP41934	ncbiId:BAH02044	ncbiId:BAH02091	ncbiId:BAH02133
ncbiId:ACP41943	ncbiId:BAH02046	ncbiId:BAH02087	ncbiId:BAH02134
ncbiId:ACP41942	ncbiId:BAH02045	ncbiId:BAH02088	ncbiId:BAH02135
ncbiId:ACP41944	ncbiId:BAH02040	ncbiId:BAH02089	ncbiId:BAH02131
ncbiId:ACP41946	ncbiId:BAH02041	ncbiId:ABY40398	ncbiId:BAH02132
ncbiId:ACP41945	ncbiId:BAH02043	ncbiId:BAH02095	ncbiId:BAH02130
ncbiId:ACP41947	ncbiId:BAH02042	ncbiId:BAH02096	ncbiId:BAH02136
ncbiId:ACP41948	ncbiId:BAH02047	ncbiId:BAH02092	
ncbiId:ACR38809	ncbiId:BAH02050	ncbiId:BAH02093	
ncbiId:ACU13094	ncbiId:BAH02051	ncbiId:BAH02094	
ncbiId:ACU13081	ncbiId:BAH02054	ncbiId:BAH02090	
ncbiId:ACU13060	ncbiId:BAH02048	ncbiId:ABY40415	
ncbiId:ACU13059	ncbiId:BAH02049	ncbiId:BAH02145	
ncbiId:ACS94566	ncbiId:BAH02052	ncbiId:BAH02144	
ncbiId:ACR19311	ncbiId:BAH02055	ncbiId:BAH02137	
ncbiId:ACR19260	ncbiId:BAH02056	ncbiId:BAH02138	
ncbiId:ACP44165	ncbiId:BAH02053	ncbiId:BAH02139	
ncbiId:ACU13087	ncbiId:BAH02060	ncbiId:BAH02140	
ncbiId:ACQ55353	ncbiId:BAH02061	ncbiId:ABY40403	
ncbiId:ACQ55352	ncbiId:BAH02062	ncbiId:BAH02141	
ncbiId:ACQ55354	ncbiId:BAH02058	ncbiId:BAH02143	
ncbiId:ACQ55355	ncbiId:BAH02059	ncbiId:BAH02146	
ncbiId:ACQ55357	ncbiId:BAH02063	ncbiId:BAH02142	
ncbiId:ACQ55356	ncbiId:BAH02065	ncbiId:ABY40413	
ncbiId:ACS72682	ncbiId:BAH02066	ncbiId:BAH02118	
ncbiId:ACX46234	ncbiId:BAH02057	ncbiId:BAH02119	
ncbiId:ACX46207	ncbiId:BAH02064	ncbiId:BAH02120	
ncbiId:ABK57102	ncbiId:BAH02073	ncbiId:BAH02123	
ncbiId:ABK57104	ncbiId:BAH02071	ncbiId:BAH02125	
ncbiId:ABK62870	ncbiId:BAH02076	ncbiId:BAH02126	
ncbiId:ABK57106	ncbiId:BAH02067	ncbiId:ABY40401	
ncbiId:ABK57100	ncbiId:BAH02068	ncbiId:BAH02121	
ncbiId:ABK57098	ncbiId:BAH02069	ncbiId:BAH02122	
ncbiId:ABK57093	ncbiId:BAH02070	ncbiId:BAH02124	
ncbiId:ABK62868	ncbiId:ABY40394	ncbiId:BAH02117	
ncbiId:BAH02031	ncbiId:ABY40406	ncbiId:BAH02097	
ncbiId:BAH02027	ncbiId:BAH02075	ncbiId:BAH02106	
ncbiId:BAH02035	ncbiId:BAH02072	ncbiId:BAH02100	
ncbiId:BAH02036	ncbiId:BAH02074	ncbiId:BAH02103	
ncbiId:BAH02032	ncbiId:ABY40408	ncbiId:BAH02104	

Oslen et al. dataset

ncbiId:ABF17978<br>ncbiId:ABF18008<br>ncbiId:ABF17996<br>ncbiId:ABF17984<br>ncbiId:ABF17985<br>ncbiId:ABF17972<br>ncbiId:ABF17961<br>ncbiId:ABF17960<br>ncbiId:ABF17954<br>ncbiId:ABF18002<br>ncbiId:ABF17979<br>ncbiId:ABF17987<br>ncbiId:ABF17962<br>ncbiId:ABF17955<br>ncbiId:ABF18003<br>ncbiId:ABF18009<br>ncbiId:ABF17986<br>ncbiId:ABF17973<br>ncbiId:ABF17963<br>ncbiId:ABF17997<br>ncbiId:ABF17980<br>ncbiId:ABF17989<br>ncbiId:ABF17988<br>ncbiId:ABF18004<br>ncbiId:ABF18010<br>ncbiId:ABF17974<br>ncbiId:ABF17964<br>ncbiId:ABF17956<br>ncbiId:ABF17998<br>ncbiId:ABF17965<br>ncbiId:ABF17981<br>ncbiId:ABF18011<br>ncbiId:ABF17999<br>ncbiId:ABF17991<br>ncbiId:ABF17975<br>ncbiId:ABF17967<br>ncbiId:ABF17957<br>ncbiId:ABF17990<br>ncbiId:ABF18005<br>ncbiId:ABF17966<br>ncbiId:ABF17982<br>ncbiId:ABF17976<br>ncbiId:ABF17969<br>ncbiId:ABF17968	ncbiId:ABF18006<br>ncbiId:ABF18000<br>ncbiId:ABF17993<br>ncbiId:ABF17992<br>ncbiId:ABF18012<br>ncbiId:ABF17983<br>ncbiId:ABF18013<br>ncbiId:ABF18001<br>ncbiId:ABF17994<br>ncbiId:ABF17977<br>ncbiId:ABF17971<br>ncbiId:ABF17970<br>ncbiId:ABF17959<br>ncbiId:ABF17995<br>ncbiId:ABF18007

Khiabanian et al. dataset

ABF17955	ABW36327	ABR28581	ABY81431	ABS53361	ABA27435
ABF17963	ABW36328	ABR28582	ABY81432	ABS53362	ABA27436
ABF17962	ABW36329	ABR28583	ABY81433	ABS53363	ABY40428
ABF17973	ABW36330	ABR28584	ABY81434	ABS53364	ABY40427
ABF17979	ABW36332	ABR28585	ABY81436	ABS53365	ABY40429
ABF17987	ABU80410	ABR28586	ABR29585	ABS53367	ABY40430
ABF17986	ABU80411	ABR28587	ABR29587	ABS53366	ABY40432
ABF17997	ABU80412	ABR28588	ABR29586	ABS53368	ABY40431
ABF18003	ABU80413	ABR28589	ABR29588	ABS53369	ABY40433
ABF18009	ABU80414	ABR28591	ABR29589	BAG49619	ABY40434
ABQ45458	ABU80415	ABU80210	ABR29590	BAG49620	ABY40435
ABQ45459	ABU80416	ABU80211	ABR29591	BAG49621	ABY40436
ABQ45460	ABU80417	ABU80212	ABR29592	BAG49622	ABB86880
ABQ45461	ABU80418	ABU80213	ABR29593	BAG49623	ABB86881
ABQ45462	ABU80419	ABU80214	ABR29594	BAG49624	ABB86884
ABQ45463	ABU80199	ABU80215	ACQ84487	BAG49625	ABB86883
ABQ45464	ABU80200	ABU80216	ACQ84489	BAG49626	ABB86885
ABQ45465	ABU80201	ABU80217	ACQ84488	BAG49627	ABB86886
ABQ45466	ABU80202	ABU80218	ACQ84490	BAG49628	ABB86887
ABQ45468	ABU80203	ABU80220	ACQ84491	ACI48760	ABB86889
ABQ45436	ABU80204	ABR28636	ACQ84492	ACI48761	ABB86888
ABQ45437	ABU80205	ABR28637	ACQ84493	ACI48762	ABB86882
ABQ45438	ABU80206	ABR28638	ACQ84494	ACI48764	ABB86900
ABQ45439	ABU80207	ABR28639	ACQ84495	ACI48765	ABB86901
ABQ45440	ABU80209	ABR28640	ACQ84496	ACI48767	ABB86902
ABQ45441	ABR28548	ABR28641	ABE27153	ACI48766	ABB86903
ABQ45442	ABR28549	ABR28642	ABE27155	ACI48768	ABB86904
ABQ45443	ABR28550	ABR28643	ABE27154	ACI48770	ABB86905
ABQ45444	ABR28551	ABR28644	ABE27156	ACI48769	ABB86906
ABQ45446	ABR28552	ABR28646	ABE27157	ABQ41895	ABB86907
ABQ45533	ABR28553	ABW36355	ABE27159	ABQ41896	ABB86909
ABQ45534	ABR28554	ABW36357	ABE27158	ABQ41897	ABB86908
ABQ45535	ABR28555	ABW36356	ABE27160	ABQ41898	ABB86870
ABQ45536	ABR28556	ABW36358	ABE27161	ABQ41899	ABB86871
ABQ45537	ABR28558	ABW36359	ABE27163	ABQ41900	ABB86872
ABQ45538	ABR15874	ABW36361	AAZ79392	ABQ41902	ABB86874
ABQ45539	ABR15875	ABW36360	AAZ79393	ABQ41901	ABB86873
ABQ45540	ABR15876	ABW36362	AAZ79395	ABQ41904	ABB86875
ABQ45541	ABR15877	ABW36363	AAZ79394	ABQ41903	ABB86876
ABQ45542	ABR15878	ABW36365	AAZ79396	ABA27429	ABB86877
ABW36322	ABR15879	ABY81426	AAZ79397	ABA27430	ABB86879
ABW36323	ABR15880	ABY81428	AAZ79398	ABA27431	ABB86878
ABW36324	ABR15881	ABY81427	AAZ79399	ABA27432	ABB86920
ABW36325	ABR15882	ABY81429	AAZ79400	ABA27433	ABB86921
ABW36326	ABR15884	ABY81430	ABS53360	ABA27434	ABB86922

ABB86923	AAL87903	AAD51243			
ABB86924	AAL87868	AAD51255			
ABB86925	AAL87932	AAD51251			
ABB86927	AAG01766	AAD51247			
ABB86926	AAG01758	AAD51239			
ABB86928	AAG01759	AAD51271			
ABB86929	AAG01761	ABA27435			
ACE78028	AAG01762	ABA27431			
ACE77927	AAG01763	ABA27430			
ACE78067	AAG01764	ABA27436			
ACE78087	AAG01765	ABA27429			
ACE78126	AAG01760	ABA27432			
ACE77948	ABD62841	ABA27434			
ACE77947	ABD62837	ABA27433			
ACE78007	ABD62839	ABB86942			
ACE77987	ABD62838	ABB86949			
ACE78027	ABD62833	ABB86948			
ACD65217	ABD62840	ABB86941			
ACD65188	ABD62835	ABB86943			
ACD65214	ABD62834	ABB86940			
ACD65216	ABD62836	ABB86947			
ACD65215	ABD62842	ABB86946			
CAC85234	ACE77942	ABB86945			
CAC86634	ACE78058	ABB86944			
CAC86633	ACE78057	ABA27435			
CAC86620	ACE77978	ABA27431			
CAC85218	ACE77977	ABA27430			
CAC86326	ACE78022	ABA27436			
CAC87396	ACE78082	ABA27429			
CAC84756	ACE78116	ABA27432			
CAC84743	ACE78002	ABA27434			
CAC87397	ACE78141	ABA27433			
AAF75997	ACE77982				
AAF75998	ACE78143				
AAF75999	ACE78084				
AAF76000	ACE78062				
AAF76001	ACE78061				
AAF76002	ACE78004				
AAF75995	ACE78120				
AAF75996	ACE78024				
AAF75994	ACE77981				
AAL87916	ACE77944				
AAL87924	AAD51259				
AAL87876	AAD51263				
AAL87884	AAD51264				
AAL87904	AAD51272				

de Silva et al. dataset

AEA04387	ABB19077	AEH04380	AAX53538	ADQ20513	ACU15840
AEA04388	ABB19078	AEH04381	AAX53539	ADQ20514	ACU15841
AEA04389	ABB19079	AEH04383	AAX53552	ADQ20515	ACU15843
AEA04390	ABB19080	AEH04384	AAX53559	ADQ20516	ACU15844
AEA04391	ABB19082	AEH04385	AAX53565	ADQ20517	ACU15847
AEA04392	ABB19151	AEH04386	ABZ91681	ADQ20518	ACR49448
AEA04393	ABB19152	AEH04387	ABZ91682	ADQ20519	ACR49449
AEA04394	ABB19153	AEH04388	ABZ91683	ADQ20520	ACR49451
AEA04395	ABB19154	AEH04389	ABZ91684	ADQ20521	ACR49452
AEA04397	ABB19155	AEH04390	ABZ91685	ADQ20523	ACR49453
ADV76653	ABB19156	ADQ20574	ABZ91686	ADA82123	ACR49454
ADV76654	ABB19157	ADQ20575	ABZ91687	ADA82124	ACR49455
ADV76655	ABB19158	ADQ20576	ABZ91688	ADA82125	ACR49456
ADV76656	ABB19159	ADQ20577	ABZ91689	ADA82126	ACR49457
ADV76657	ABB19161	ADQ20578	ABZ91690	ADA82127	ACR49458
ADV76658	ACF25611	ADQ20579	ABR37341	ADA82128	ACJ26271
ADV76659	ACF25612	ADQ20580	ABR37342	ADA82129	ACJ26272
ADV76660	ACF25613	ADQ20581	ABR37343	ADA82130	ACJ26274
ADV76661	ACF25614	ADQ20582	ABR37344	ADA82131	ACJ26275
ADV76663	ACF25615	ADQ20584	ABR37345	ADA82133	ACJ26276
ABB87377	ACF25616	BAF46444	ABR37346	ADQ26662	ACJ26277
ABB87378	ACF25617	BAF46445	ABR37347	ADQ26663	ACJ26278
ABB87379	ACF25618	BAF46446	ABR37348	ADQ26664	ACJ26279
ABB87380	ACF25620	BAF46447	ABR37349	ADQ26665	ACJ26280
ABB87381	ACF25621	BAF46448	ABR37351	ADQ26666	ACJ26281
ABB87383	ABB88183	BAF46449	ADP06894	ADQ26667	ACY78046
ABB87382	ABB88184	BAF46450	ADP06895	ADQ26668	ACY78047
ABB87384	ABB88185	BAF46451	ADP06896	ADQ26669	ACY78049
ABB87385	ABB88186	BAF46452	ADP06897	ADQ26670	ACY78050
ABB87387	ABB88187	BAF46453	ADP06898	ADQ26672	ACY78051
ABB87042	ABB88188	ABB18069	ADP06899	ACP50660	ACY78052
ABB87044	ABB88189	ABB18070	ADP06900	ACP50661	ACY78053
ABB87043	ABB88190	ABB18071	ADP06901	ACP50663	ACY78054
ABB87045	ABB88191	ABB18072	ADP06902	ACP50664	ACY78055
ABB87046	ABB88193	ABB18073	ADP06904	ACP50665	ACY78056
ABB87048	ABS70431	ABB18074	ACV86891	ACP50666	ADQ92669
ABB87047	ABS70432	ABB18075	ACV86892	ACP50667	ADQ92670
ABB87049	ABS70433	ABB18076	ACV86894	ACP50668	ADQ92671
ABB87050	ABS70434	ABB18077	ACV86895	ACP50669	ADQ92672
ABB87052	ABS70435	ABB18079	ACV86896	ACP50670	ADQ92673
ABB19072	ABS70437	AAX53504	ACV86897	ACU15833	ADQ92674
ABB19073	ABS70428	AAX53511	ACV86898	ACU15835	ADQ92675
ABB19074	ABS70429	AAX53512	ACV86899	ACU15836	ADQ92676
ABB19075	ABS70430	AAX53525	ACV86893	ACU15837	ADQ92677
ABB19076	ABS70427	AAX53531	ACV86890	ACU15839	ADQ92679

ABY50570	ACU46691	ABB87918	ACZ48237	ACU15579	ADD21454
ABY50571	ACU46697	ABB87924	ACZ47407	ACU15581	ADD21446
ABY50572	ACU46709	ABB87923	ADQ92622	ACU15583	ADD21451
ABY50573	ACU46720	ABB87925	ADQ92619	ACU15570	ADD21450
ABY50574	ACA42428	ABB87926	ADQ92621	ACU15572	ADD21453
ABY50575	ACA42429	ABB87928	ADQ92620	ACU15574	ADD21449
ABY50576	ACA42431	ABB87922	ADQ92625	ACZ05887	AED99980
ABY50577	ACA42430	ABB87919	ADQ92615	ACZ05812	AED99983
ABY50578	ACA42432	AAD51922	ADQ92617	ACZ05811	AED99987
ABY50579	ACA42433	AAD51927	ADQ92616	ACZ05840	AED99978
ACJ26370	ACA42434	AAD51928	ADQ92623	ACZ05839	AED99981
ACJ26371	ACA42435	AAD51929	ADQ92618	ACZ05859	AED99982
ACJ26373	ACA42436	AAD51924	ACZ05870	ACZ05901	AED99985
ACJ26374	ACA42437	AAD51925	ACZ05926	ACZ05915	AED99984
ACJ26375	ABV55857	AAD51930	ACZ05884	ACZ05873	AED99979
ACJ26376	ABV55858	AAD51931	ACZ05805	ACZ05929	AED99986
ACJ26379	ABV55859	AAD51926	ACZ05834	ACU44783	ADG59576
ACJ26380	ABV55860	AAD51923	ACZ05833	ACU44782	ADD21452
ACJ26377	ABV55861	AAD37782	ACZ05912	ACX53682	ADD21448
ACJ26378	ABV55862	BAG13020	ACZ05898	ACX53685	ADD21447
ADU53077	ABV55863	BAF41921	ACZ05856	ACX53679	
ADU53078	ABV55864	BAF41912	ACZ05806	ACX53687	
ADU53079	ABV55866	BAF41920	ADW80461	ACX53686	
ADU53080	ABV55865	BAF41916	ADW80460	ACX53680	
ADU53081	ABQ44394	BAF41917	ADW80462	ACX53684	
ADU53082	ABQ44395	BAF41914	ADW80465	ACX53683	
ADU53083	ABQ44396	BAF41915	ADW80464	CAC85234	
ADU53084	ABQ44397	BAF41911	ADW80459	CAC86634	
ADU53085	ABQ44398	ACZ36596	ADW80466	CAC86633	
ADU53087	ABQ44399	ACZ36883	ADW80463	CAC86620	
BAI48888	ABQ44400	ACZ36654	ADW80467	CAC85218	
BAI48889	ABQ44401	ACZ36653	ADW80469	CAC86326	
BAI48891	ABQ44402	ACZ36717	ACU15374	CAC87396	
BAI48892	ABQ44404	ACZ36759	ACU15377	CAC84756	
BAI48893	ACQ73419	ACZ36814	ACU15376	CAC84743	
BAI48894	ACQ73420	ACZ36813	ACU15378	CAC87397	
BAI48895	ACQ73421	ACZ36506	ACU15381	ABJ15722	
BAI48896	ACQ73414	ACZ36551	ACU15372	ABJ15724	
BAI48897	ACQ73413	ACZ45525	ACU15375	ABJ15723	
BAI48898	ACQ73415	ACZ46310	ACU15371	ABJ15725	
ACU46656	ACQ73416	ACZ47408	ACU15379	ABJ15717	
ACU46655	ACQ73418	ACZ45928	ACU15373	ABJ15716	
ACU46667	ACQ73417	ACZ45202	ACU15571	ABJ15719	
ACU46673	ACQ73412	ACZ46692	ACU15575	ABJ15720	
ACU46679	ABB87920	ACZ48589	ACU15578	ABJ15721	
ACU46680	ABB87921	ACZ48238	ACU15577	ABJ15718	

Niranjan et al. dataset

ACP44151	ABY84689	BAH02147	ABR29600	ABS50121	ABR28619
ACP44153	ABY84690	BAH02148	ABR29601	ABS50122	ABR28620
ACP44152	ABY84691	BAH02149	ABR29602	ABS50123	ABR28621
ACP44154	ABY84692	BAH02150	ABR29603	ABS50124	ABR28622
ACP44155	ABY84693	BAH02151	ABR29604	ABS50125	ABR28624
ACP44156	BAH02077	BAH02152	ABW86596	ABS50126	BAG49739
ACP44157	BAH02078	BAH02153	ABW86598	ABS50127	BAG49740
ACP44158	BAH02079	BAH02154	ABW86597	ABS50128	BAG49741
ACQ76318	BAH02080	BAH02155	ABW86599	ABS50129	BAG49742
ACT36632	BAH02081	BAH02156	ABW86600	ABS50130	BAG49743
ACP41935	BAH02082	ABR29615	ABW86601	ABR15819	BAG49744
ACP41936	BAH02083	ABR29617	ABW86602	ABR15820	BAG49745
ACP41938	BAH02084	ABR29616	ABW86603	ABR15821	BAG49746
ACP41937	BAH02086	ABR29618	ABW86604	ABR15822	BAG49747
ACP41939	BAH02085	ABR29619	ABW86605	ABR15823	BAG49748
ACP41940	BAH02067	ABR29620	ABX58646	ABR15824	ABR29565
ACP41941	BAH02068	ABR29621	ABX58648	ABR15825	ABR29567
ACP52563	BAH02069	ABR29622	ABX58647	ABR15826	ABR29566
ACP52562	BAH02070	ABR29623	ABX58649	ABR15827	ABR29568
ACP52564	BAH02071	ABR29624	ABX58650	ABR15829	ABR29569
AGI54699	BAH02072	ABY81426	ABX58651	ABU80420	ABR29571
AGI54701	BAH02073	ABY81428	ABX58652	ABU80421	ABR29570
AGI54702	BAH02074	ABY81427	ABX58653	ABU80422	ABR29572
AGI54704	BAH02075	ABY81429	ABX58654	ABU80423	ABR29573
AGI54703	BAH02076	ABY81430	ABX58656	ABU80424	ABR29574
AGI54706	BAH02047	ABY81431	ABW71521	ABU80426	ACI26604
AGI54705	BAH02048	ABY81432	ABW71523	ABU80425	ACI26606
AGI54707	BAH02049	ABY81433	ABW71522	ABU80427	ACI26605
AGI54708	BAH02050	ABY81434	ABW71524	ABU80428	ACI26607
AGI54709	BAH02051	ABY81436	ABW71525	ABU80429	ACI26608
ABB86880	BAH02052	ABS50111	ABW71527	ABR29575	ACI26609
ABB86881	BAH02053	ABS50113	ABW71526	ABR29576	ACI26610
ABB86884	BAH02054	ABS50112	ABW71528	ABR29577	ACI26611
ABB86883	BAH02055	ABS50114	ABW71529	ABR29578	ACI26612
ABB86885	BAH02056	ABS50115	ABW71530	ABR29579	ACI26613
ABB86886	BAH02057	ABS50116	ABR29605	ABR29581	ABR28647
ABB86887	BAH02058	ABS50117	ABR29607	ABR29580	ABR28648
ABB86889	BAH02059	ABS50118	ABR29606	ABR29582	ABR28649
ABB86888	BAH02060	ABS50119	ABR29608	ABR29583	ABR28650
ABB86882	BAH02061	ABS50120	ABR29609	ABR29584	ABR28651
ABY84684	BAH02062	ABR29595	ABR29610	ABR28614	ABR28652
ABY84686	BAH02063	ABR29597	ABR29611	ABR28615	ABR28653
ABY84685	BAH02064	ABR29596	ABR29612	ABR28616	ABR28654
ABY84687	BAH02065	ABR29598	ABR29613	ABR28617	ABR28655
ABY84688	BAH02066	ABR29599	ABR29614	ABR28618	ABR28657

BAH02157	ABW36351	ABR28673	ABU80266	ABR15871	ABY51220
BAH02158	ABW36352	ABR28675	ABU80267	ABR15873	ABY51221
BAH02159	ABW36354	ABR28674	ABU80268	ABU80199	ABY51222
BAH02160	ABR28658	ABR28676	ABU80269	ABU80200	ABY51223
BAH02161	ABR28659	ABR28677	ABU80270	ABU80201	ABY51225
BAH02162	ABR28660	ABR28679	ABU80271	ABU80202	ABR28570
BAH02163	ABR28661	BAH02027	ABU80272	ABU80203	ABR28571
BAH02164	ABR28662	BAH02028	ABU80273	ABU80204	ABR28572
BAH02165	ABR28664	BAH02029	ABU80275	ABU80205	ABR28573
BAH02166	ABR28663	BAH02030	ABR28691	ABU80206	ABR28574
ACH69547	ABR28665	BAH02031	ABR28692	ABU80207	ABR28575
ACH69548	ABR28666	BAH02032	ABR28693	ABU80209	ABR28576
ACH69549	ABR28668	BAH02033	ABR28694	ABR28548	ABR28577
ACH69550	ABB86900	BAH02034	ABR28695	ABR28549	ABR28578
ACH69552	ABB86901	BAH02035	ABR28696	ABR28550	ABR28580
ACH69553	ABB86902	BAH02036	ABR28697	ABR28551	ABW86585
ACH69556	ABB86903	BAH02037	ABR28698	ABR28552	ABW86586
ACH69557	ABB86904	BAH02038	ABR28699	ABR28553	ABW86587
ACH69554	ABB86905	BAH02039	ABR28701	ABR28554	ABW86588
ACH69555	ABB86906	BAH02040	ABD95712	ABR28555	ABW86589
ABW36333	ABB86907	BAH02041	ABD95713	ABR28556	ABW86590
ABW36335	ABB86909	BAH02042	ABD95714	ABR28558	ABW86591
ABW36334	ABB86908	BAH02043	ABD95715	ABR15874	ABW86592
ABW36336	ABB86870	BAH02044	ABD95716	ABR15875	ABW86593
ABW36337	ABB86871	BAH02045	ABD95717	ABR15876	ABW86595
ABW36339	ABB86872	BAH02046	ABD95718	ABR15877	ACA96530
ABW36338	ABB86874	ACA25337	ABD95719	ABR15878	ACA96531
ABW36340	ABB86873	ACA25340	ABD95720	ABR15879	ACA96532
ABW36341	ABB86875	ACA25339	ABD95721	ABR15880	ACA96533
ABW36343	ABB86876	ACA25341	ABX58657	ABR15881	ACA96534
ABB86930	ABB86877	ACA25342	ABX58658	ABR15882	ACA96535
ABB86931	ABB86879	ACA25344	ABX58659	ABR15884	ACA96536
ABB86932	ABB86878	ACA25343	ABX58660	ABR28559	ACA96537
ABB86934	ABW36355	ACA25345	ABX58661	ABR28560	ACA96538
ABB86933	ABW36357	ACA25346	ABX58662	ABR28561	ACA96540
ABB86935	ABW36356	ACA25347	ABX58663	ABR28562	ABR28713
ABB86936	ABW36358	ABR28537	ABX58664	ABR28563	ABR28715
ABB86937	ABW36359	ABR28538	ABX58665	ABR28564	ABR28714
ABB86939	ABW36361	ABR28539	ABX58667	ABR28565	ABR28716
ABB86938	ABW36360	ABR28540	ABR15863	ABR28566	ABR28717
ABW36344	ABW36362	ABR28541	ABR15864	ABR28567	ABR28719
ABW36345	ABW36363	ABR28542	ABR15865	ABR28569	ABR28718
ABW36346	ABW36365	ABR28543	ABR15866	ABY51215	ABR28720
ABW36347	ABR28669	ABR28544	ABR15867	ABY51216	ABR28721
ABW36348	ABR28671	ABR28545	ABR15868	ABY51217	ABR28723
ABW36350	ABR28670	ABR28547	ABR15869	ABY51218	ABR29585
ABW36349	ABR28672	ABU80265	ABR15870	ABY51219	ABR29587

ABR29586	ABS49964	ACO25147	ACD65215	AAV30840	ABV60438
ABR29588	ABR28735	ACO25150	ABS49936	ACN39452	ABV60437
ABR29589	ABR28737	ACO25149	ABS49942	ACN39415	ABV60440
ABR29590	ABR28736	ACO25141	ABS49940	ACN39478	ABQ58891
ABR29591	ABR28738	AAB50959	ABS49933	ACN39510	ABQ58915
ABR29592	ABR28739	AAB50975	ABS49932	ACN39390	ABQ58886
ABR29593	ABR28741	AAB50971	ABS49937	ACN39389	ABQ58921
ABR29594	ABR28740	AAB50983	ABS49935	ACN39494	ABQ58906
ACQ84487	ABR28742	AAB51007	ABS49934	ACN39357	ABQ58905
ACQ84489	ABR28743	AAB50973	ABS49939	ACN39431	ABQ58877
ACQ84488	ABR28745	AAB52713	ABS49938	ACN39358	ABQ58876
ACQ84490	ABR28746	AAB50969	ABB86942	AAO53052	ABQ58871
ACQ84491	ABR28747	AAB51008	ABB86949	AAO52948	ABQ58901
ACQ84492	ABR28748	AAB50984	ABB86948	AAO53026	ABA87105
ACQ84493	ABR28749	ACE78028	ABB86941	AAO52923	ABI33783
ACQ84494	ABR28750	ACE77927	ABB86943	AAO53000	ABI33813
ACQ84495	ABR28751	ACE78067	ABB86940	AAO52974	ABI33800
ACQ84496	ABR28752	ACE78087	ABB86947	AAO52872	ABI33772
ABX58668	ABR28753	ACE78126	ABB86946	AAO52897	ABI33791
ABX58669	ABR28754	ACE77948	ABB86945	ABL07925	ABI33771
ABX58670	ABR28756	ACE77947	ABB86944	ABJ96630	ABI33805
ABX58671	ABR28757	ACE78007	ABU80283	ABJ96858	ABA87102
ABX58672	ABR28759	ACE77987	ABU80286	ABL08212	ABC69149
ABX58673	ABR28758	ACE78027	ABU80278	ABL08211	ABC74402
ABX58674	ABR28760	ACE78030	ABU80281	ABL08529	ABC74404
ABX58675	ABR28761	ACE78127	ABU80282	ABL08378	ABC74410
ABX58676	ABR28762	ACE78008	ABU80277	ABL08982	ABC73060
ABX58678	ABR28763	ACE77928	ABU80279	ABL08816	ABC74414
ACK99410	ABR28764	ACE78089	ABU80280	ABL08815	ABC74393
ACK99411	ABR28765	ACE77988	ABU80276	AAX53521	ABC74392
ACK99412	ABR28767	ACE78068	ABU80284	AAX53522	ABC74403
ACK99413	ACO24988	ACE77949	ABK00133	CAQ63500	ABC74409
ACK99414	ACO24980	ACE77950	ABK00134	CAQ63506	ACJ26362
ACK99415	ACO24982	ACE78029	ABK00135	CAQ63503	ACJ26365
ACK99416	ACO24984	ABR28646	ABK00137	CAQ63504	ACJ26367
ACK99417	ACO24985	ABR28641	ABK00136	CAQ63498	ACJ26366
ACK99418	ACO24987	ABR28643	ABK00140	CAQ63501	ACJ26363
ACK99420	ACO24986	ABR28636	ABK00139	CAQ63499	ACJ26369
ABS49954	ACO24989	ABR28640	ABK00141	CAQ63502	ACJ26359
ABS49955	ACO24983	ABR28639	ABK00138	CAQ63505	ACJ26368
ABS49956	ACO25140	ABR28637	AAV30836	ABV60446	ACJ26364
ABS49957	ACO25143	ABR28638	AAV30837	ABV60445	ACJ26360
ABS49958	ACO25144	ABR28642	AAV30838	ABV60444	ACJ26371
ABS49959	ACO25145	ABR28644	AAV30839	ABV60439	ACJ26373
ABS49960	ACO25146	ACD65217	AAV30841	ABV60441	ACJ26374
ABS49961	ACO25148	ACD65188	AAV30842	ABV60442	ACJ26370
ABS49962	ACD65216	ACD65214	AAV30843	ABV60443	ACJ26378

ACJ26377<br>ACJ26375<br>ACJ26376<br>ACJ26380<br>ACJ26379<br>ACB70721<br>ACB70723<br>ACB70724<br>ACB70725<br>ACB70726<br>ACB70728<br>ACB70727<br>ACB70730<br>ACB70729<br>ACB70720<br>ACD64986<br>ACD62245<br>ACD62239<br>ACD62241<br>ACD62242<br>ACD64987<br>ACD64988<br>ACD62243<br>ACD62244<br>ACD62240<br>ACG58516<br>ACG58519<br>ACG58526<br>ACG58518<br>ACG58517<br>ACG58521<br>ACG58523<br>ACG58524<br>ACG58520<br>ACG58522<br>ABJ52574<br>ABJ52577<br>ABJ52576<br>ABJ52578<br>ABJ52575<br>ABJ52573<br>ABJ52572<br>ABJ80586<br>ABJ80587<br>ABJ80592<br>ABJ80590	ABJ80589<br>ABJ80591<br>ABJ80588<br>ABK00083<br>ABK00084<br>ABK00086<br>ABK00085<br>ABJ52567<br>ABJ52570<br>ABJ52571<br>ABJ52566<br>ABJ52565<br>ABJ52568<br>ABJ52569<br>BAE07152<br>BAE07158<br>BAE07159<br>BAE07155<br>BAE07153<br>BAE07157<br>BAE07154<br>BAE07160<br>BAE07161<br>BAE07156<br>AAT70554<br>AAT70555<br>AAT70643<br>AAT73287<br>AAT73420<br>AAT73421<br>AAT74500<br>AAT73341<br>AAT73509<br>AAT73563				
